# Opposing Effects of LRIG1 and LRIG3 on EGFR Maintain Intestinal Homeostasis and Mitigate Likelihood of Colonic Neoplasia

**DOI:** 10.1016/j.jcmgh.2026.101873

**Published:** 2026-08-31

**Authors:** Matthew E. Bechard, Zhengyi Chen, Ping Zhao, Radhika Aramandla, Yilin Yang, Alan J. Simmons, M. Kay Washington, Jeffery L. Franklin, Ken S. Lau, Robert J. Coffey

**Affiliations:** 1 Department of Medicine, Vanderbilt University Medical Center, Nashville, Tennessee; 2 Epithelial Biology Center, Vanderbilt University Medical Center, Nashville, Tennessee; 3 Chemical and Physical Biology Program, Vanderbilt University, Nashville, Tennessee; 4 Department of Cell and Developmental Biology, Vanderbilt University School of Medicine, Nashville, Tennessee; 5 Department of Pathology, Microbiology, and Immunology, Vanderbilt University Medical Center, Nashville, Tennessee; 6 Center for Computational Systems Biology, Vanderbilt University, Nashville, Tennessee

**Keywords:** Colorectal Cancer, EGFR, Homeostasis, Intestinal Crypt, Intestinal Stem Cells, Lrig1, Lrig3

## Abstract

**Background & Aims:**

Epidermal growth factor receptor signaling maintains intestinal homeostasis by modulating proliferation and differentiation within the stem cell compartment, with excess epidermal growth factor receptor (EGFR) signaling predisposing to neoplasia. EGFR protein levels are central to EGFR function. These levels are negatively regulated by leucine-rich repeats and immunoglobulin-like domains 1 (LRIG1), which accelerates receptor internalization and degradation, supporting a tumor suppressor role for LRIG1. Leucine-rich repeats and immunoglobulin-like domains 3 (LRIG3) is a less-studied family member that has been reported, in a context-dependent manner, to both cooperate with and oppose the effects of LRIG1 on epidermal growth factor receptor.

**Methods:**

To examine the function of LRIG3 relative to LRIG1 on EGFR levels and downstream signaling *in vivo*, we generated an *Lrig3* floxed allele and intercrossed these mice with *Lrig1*^CreERT2^ mice that are *Lrig1* null in the homozygous state. This allowed us to assess the effects of *Lrig3* loss in *Lrig1*-null intestinal cells. We also tested how LRIG3 depletion in *Lrig1*-expressing cells affects colorectal tumors in *Lrig1*^CreERT2/+^; *Apc*^fl/+^ mice.

**Results:**

Unlike the heightened EGFR and Wnt activity caused by *Lrig1* loss, loss of *Lrig3* in *Lrig1*^Null^ cells markedly reduced activity in both pathways. In this setting, a widening of the intestinal crypt base was observed with the appearance of intermediate-lineage secretory cells at the expense of stem cells. In marked contrast to multiple distal colonic tumors in *Lrig1*^CreERT2/+^; *Apc*^fl/+^ mice, deletion of a single *Lrig3* allele in *Lrig1* haploinsufficient mice decreased EGFR signaling and markedly reduced colonic tumor formation.

**Conclusions:**

These findings indicate that LRIG3 and LRIG1 oppose one another to balance EGFR signaling, maintain intestinal homeostasis, and minimize the likelihood of colonic neoplasia.

## Graphical abstract

**Figure:**
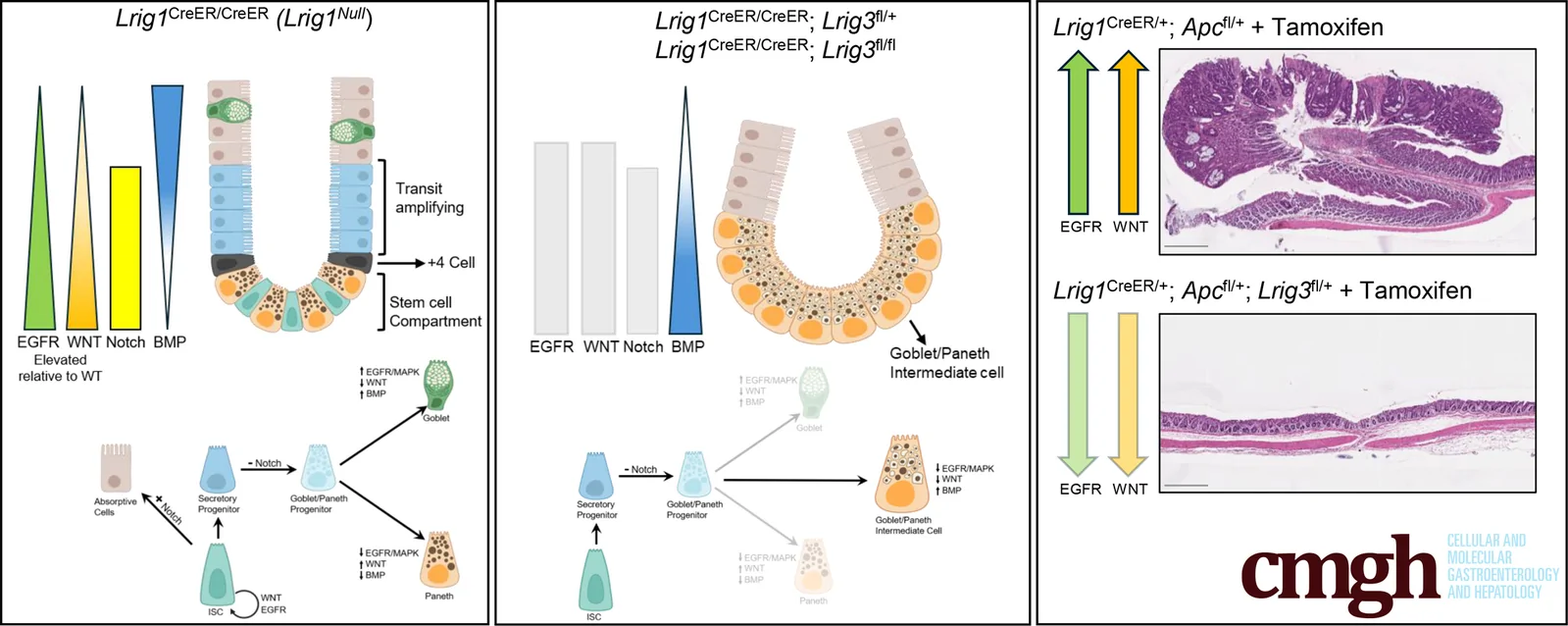

**Summary**

The antagonistic relationship between LRIG1 and LRIG3 works to balance and regulate EGFR signaling along with other downstream signaling pathways critical to maintaining intestinal crypt homeostasis. Maintaining this balance also plays a role in mitigating colonic tumor formation.

**What You Need to Know**

**Background**

The tumor suppressor LRIG1 is a negative regulator of EGFR that marks a distinct intestinal stem cell population. LRIG3, also expressed in intestinal stem cells, regulates EGFR, but it is unknown whether it cooperates with or opposes LRIG1.

**Impact**

The work presented here shows that LRIG1 and LRIG3 oppose one another to balance EGFR signaling, maintaining intestinal stem cell homeostasis and mitigating colorectal tumor formation and progression.

**Future Directions**

This work presents valuable insight and tools to further study the antagonistic relationship between LRIG1 and LRIG3 to better understand crypt homeostasis and potentially critical mechanisms related to colorectal tumor development and progression.

Under homeostatic conditions, the mouse intestinal epithelium undergoes continuous renewal, with most cells being replaced every 5 days.^1^ In response to damage, the intestinal epithelium is capable of rapid regeneration and renewal.^1^ These dynamic processes necessitate maintaining a well-regulated balance between stem cell proliferation and differentiation. *Lgr5* marks a continuously proliferative, symmetrically dividing population of crypt–base columnar cells that serve as the homeostatic stem cell population.^2^ A marker of a less proliferative stem cell population located at the crypt base is the single-pass transmembrane leucine-rich repeats and immunoglobulin-like domains 1 (LRIG1) protein.^3^^,^^4^ In addition to marking intestinal stem cells (ISCs), LRIG1 is a pan-ERBB negative regulator.^3^^,^^4^ LRIG1 interacts with epidermal growth factor receptor (EGFR) (ERBB1) through its extracellular domain. When LRIG1 binds EGFR, it promotes EGFR internalization and subsequent ubiquitin-mediated degradation by recruiting CBL, an E3 ubiquitin ligase.^5^ The net effect of this interaction is to reduce EGFR signaling.^4^, ^5^, ^6^ It should be noted that LRIG1 is able to interact with other ERBBs and other receptor tyrosine kinases (RTKs) like MET and RET.^7^^,^^8^

Mice with global deletion of *Lrig1* exhibit strain-dependent phenotypes. Deletion of *Lrig1* in C57BL/6 mice is embryonically lethal, and deletion in mixed-strain C57BL/6 × 129 mice leads to spontaneous duodenal tumors that exhibit gastric metaplasia^3^; in this setting, EGFR appears to be the major LRIG1 target as the genetic elimination of *Egfr* significantly attenuates this tumor phenotype.^9^ *Lrig1*-null mice also display markedly delayed small intestinal transit due to the loss of LRIG1 in 2 distinct populations of interstitial cells of Cajal.^10^ In contrast, FVB/N mice with global deletion of *Lrig1* survive until sacrifice at postnatal day 10 due to markedly dilated intestines, which have been attributed to crypt hyperplasia and expansion of the ISC compartment as a result of increased EGFR signaling.^11^

Along with LRIG1, leucine-rich repeats and immunoglobulin-like domains 3 (LRIG3) is another Lrig family member that is highly expressed in the gastrointestinal tract but has been much less extensively studied.^12^^,^^13^ LRIG3 is more broadly expressed along the vertical intestinal crypt gradient, relative to LRIG1, which is more restricted to the bottom of the crypt.^3^^,^^13^^,^^14^ Germline deletion of *Lrig3* is nonlethal but results in changes in inner ear architecture, cardiac hypertrophy, and reduced liver fat accumulation.^15^, ^16^, ^17^ *Lrig3*-deficient mice also exhibit ileal dilation and elongated colons^13^^,^^17^ and an increased number of stem, progenitor, and deep secretory cells (DSCs) in colonic crypts.^13^ Recent studies have implicated the dysregulation of *Lrig3* in the progression of several human cancers, such as glioblastoma, hepatocellular, prostate, and colorectal cancers.^18^, ^19^, ^20^, ^21^

Data are conflicting regarding LRIG3 function(s), with evidence that it both cooperates with and opposes LRIG1 in a context-dependent manner. During inner ear development, deletion of *Lrig1* and *Lrig3* was required to disrupt inner ear morphogenesis, strongly suggesting redundant roles.^22^ Similarly, LRIG1 and LRIG3 were shown to functionally overlap in their ability to negatively regulate RET receptor signaling during axonal growth and epidermal innervation.^23^ In addition, LRIG1 and LRIG3 act redundantly to promote bone morphogenic protein (BMP) signaling in regulating lipid metabolism.^24^^,^^25^ Conversely, several studies note context-dependent opposing functions for LRIG1 and LRIG3. For GDF7 signaling, LRIG1 was shown to be inhibitory, whereas LRIG3 enhanced signaling.^24^ Moreover, although LRIG1 has a well-documented negative role in regulating ERBBs, LRIG3 was shown to both stabilize EGFR and promote its expression.^26^^,^^27^ Of note, in contrast to LRIG1, LRIG3 lacks a CBL-binding domain. In addition, LRIG1 reportedly has a destabilizing effect on LRIG3.^27^ These latter studies suggest a mechanism by which the opposing effects of LRIG1 and LRIG3 are critical for regulation of EGFR signaling.

Given the vital role of EGFR signaling during crypt homeostasis, we sought to determine whether LRIG3 cooperates with or opposes LRIG1’s effects on EGFR regarding crypt homeostasis regulation. To accomplish this, we utilized an *Lrig1*^CreERT2^ allele to delete *Lrig3* in *Lrig1*-expressing cells. Because the *Lrig1*^CreERT2^ allele is a *Lrig1*^Null^ allele,^3^ we were able to observe the effect of LRIG3 loss in the absence of LRIG1. Here, we describe the *in vivo* effects of LRIG3 loss in *Lrig1*-expressing cells that are *Lrig1*-null as they relate to intestinal homeostasis and colonic neoplasia.

## Results

### *Lrig3* Deletion in *Lrig1*^Null^ Cells Disrupts Crypt Homeostasis, Resulting in Crypt Death

Our prior studies have focused on LRIG1 and its role as a negative regulator of EGFR in the intestinal epithelium.^3^^,^^9^^,^^28^ In contrast to *Lrig1* deletion, assessment of intestinal tissue of a global *Lrig3* deletion shows a mild phenotype.^13^ *Lrig3*^−/−^ mice exhibit ileal dilation and have longer colons with a slightly increased colonic mucosal area.^13^ To investigate the expression of *Lrig1* and *Lrig3* in the intestinal epithelium of wild-type (WT) mice, we analyzed a single-cell RNA sequencing (scRNA-seq) colonic tissue dataset recently generated by our group and a publicly available small intestinal dataset.^29^ Corroborating earlier work,^13^ *Lrig1* expression is mostly segregated to ISCs relative to *Lrig3*, which has a broader expression pattern (Figure 1*A*). Publicly available human scRNA-seq datasets^30^^,^^31^ showed similar results (Figure 1*B*). To investigate the role of LRIG3 in relation to LRIG1 more directly, we designed a mouse line that allows the inducible deletion of *Lrig3* in *Lrig1*^*+*^ ISCs by generating a *Lrig3* flox allele with loxP sites flanking exons 4 to 12 (Figure 1*C*). Given previous work suggesting functional redundancy,^23^, ^24^, ^25^ it was important to investigate the effect of *Lrig3* loss in *Lrig1*-expressing cells that no longer have LRIG1 protein. Because the CreERT2 coding sequence replaces that of *Lrig1*, the *Lrig1*^CreERT2^ allele is a *Lrig1*^Null^ allele.^3^ We therefore crossed *Lrig1*^CreERT2^ with *Lrig3*^fl^ mice to generate *Lrig1*^CreERT2/CreERT2^; *Lrig3*^fl/fl^ mice, enabling the inducible deletion of both alleles of *Lrig3* in the absence of LRIG1 protein under homeostatic conditions. The *Lrig1*^CreERT2/CreERT2^ allele will henceforth be referred to as *Lrig1*^Null^. Initially, deletion of both alleles of *Lrig3* in *Lrig1*^+^ cells was induced via intraperitoneal (i.p.) injection of 3 consecutive daily 2.5 mg doses of tamoxifen. Using this injection regimen proved lethal, with all mice dying by day 6 (N *=* 3 mice). As a result, we reduced the daily dose of tamoxifen to 2 mg and examined the mice 4 days after the last tamoxifen injection. Assessment of small intestinal and colonic crypts by immunofluorescence (IF) showed a drastic decrease in LRIG3 IF relative to both WT and corn oil–treated controls in a pattern consistent with its inducible deletion via the *Lrig1*^CreERT2^ allele (Figure 2*A*). Hybridization chain reaction-fluorescence in situ hybridization (HCR-FISH) showed the expected loss of Lrig3 messenger RNA levels in tamoxifen-treated *Lrig1*^Null^; *Lrig3*^fl/fl^ mice relative to corn oil–treated controls (Figure 2*B*). Although high doses of tamoxifen (>250 mg/kg/day, ∼5–8 mg per day) lead to weight loss, shortening of the small intestine, and negative effects on ISC proliferation and differentiation, lower doses of tamoxifen (∼50 mg/kg/day, ∼1–2 mg per day) have minimal effects on intestinal tissue or ISCs.^32^ Corroborating this, treatment of WT mice with 3 consecutive daily doses of 2 mg of tamoxifen showed no observable effect on the gross morphology of intestinal tissue, relative to untreated WT mice (Figure 3). Tamoxifen treatment alone also had no effect on either the expression pattern of *Lrig3* or the overall level of LRIG3 protein (Figure 2*A*). We did, however, observe a dramatic loss of small intestinal and colonic crypts in tamoxifen-treated *Lrig1*^Null^; *Lrig3*^fl/fl^ mice relative to controls (Figures 3 and 4*A*). Quantification of these findings showed a 69.3% decrease in the number of small intestinal crypts and a 39.1% decrease in the number of colonic crypts (Figure 4*C*). To determine if crypt loss was due to cell death, small intestinal and colonic tissues were analyzed using a terminal deoxynucleotidyl transferase dUTP nick end labeling (TUNEL) assay. Under homeostatic conditions, the crypt epithelium of corn oil–treated *Lrig1*^Null^; *Lrig3*^fl/fl^ small intestinal and colonic tissue samples was 0.28% ± 0.07% and 0.21% ± 0.05% TUNEL positive, respectively. However, TUNEL-positive cells in the tamoxifen-treated mice increased to 3.1% ± 0.62% in the small intestine and 0.87% ± 0.12% in the colon (Figures 4*B* and 5*A*). Cell death was concentrated in the mid to upper regions of the intestinal crypt epithelium (Figure 5*A*). We also observed an increase in the amount of γ-H2Ax^+^ cells in the crypts of tamoxifen-treated *Lrig1*^Null^; *Lrig3*^fl/fl^ mice (Figure 5*B*). These data show that increased cell death following the homozygous deletion of *Lrig3* in *Lrig1*^Null^ ISCs contributed significantly to the loss of intestinal crypts.

**Figure 1:**
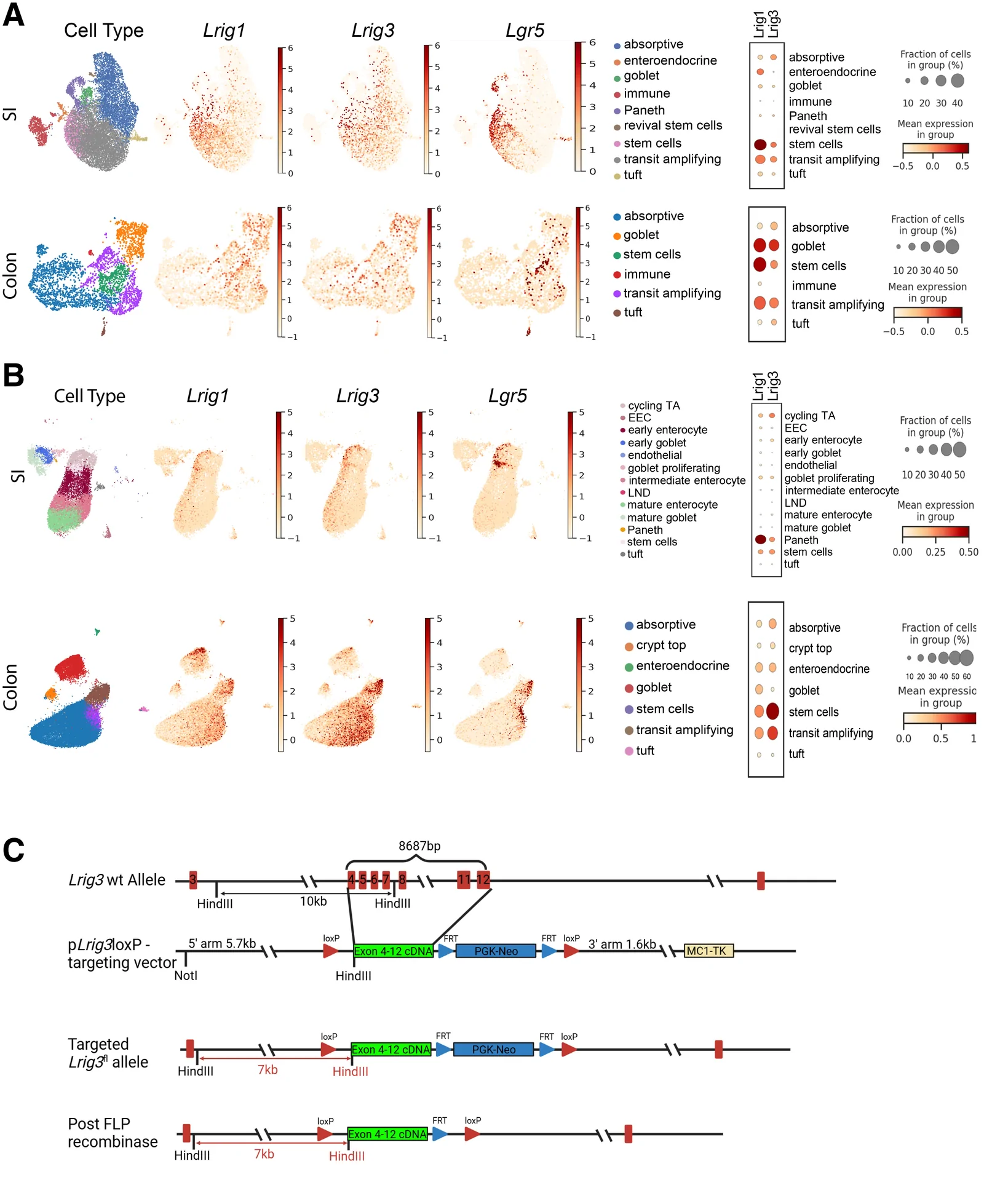
***Lrig1* and *Lrig3* expression in mouse and human intestinal tissue.** UMAP analysis indicating cell-type assignments for clusters generated based on canonical (*A*) mouse and (*B*) human gene markers, colored by gene expression levels of *Lrig1*, *Lrig3*, and *Lgr5*, with corresponding dot plots indicating the mean expression level of *Lrig1* and *Lrig3* in each of the cell types listed. (*C*) Schematic detailing generation of *Lrig3*^fl/fl^ mice. To generate the *Lrig3*^fl^ allele, the *129 Lrig3* BAC clone was obtained from the Sanger Institute, and BAC recombineering was used to construct a targeting vector that placed LoxP sites 5′ and 3′ of exons 4–12 and a PGK-Neo cassette. Homologous recombination was used to modify the native *Lrig3* locus in embryonic stem cells, which were injected into blastocyst stage 129 mouse embryos by the Transgenic Mouse/ES Cell Shared Resource at Vanderbilt University. The resulting chimeric mice were bred to *FlpE* mice to remove the PGK-Neo cassette. SI, small intestine.

**Figure 2:**
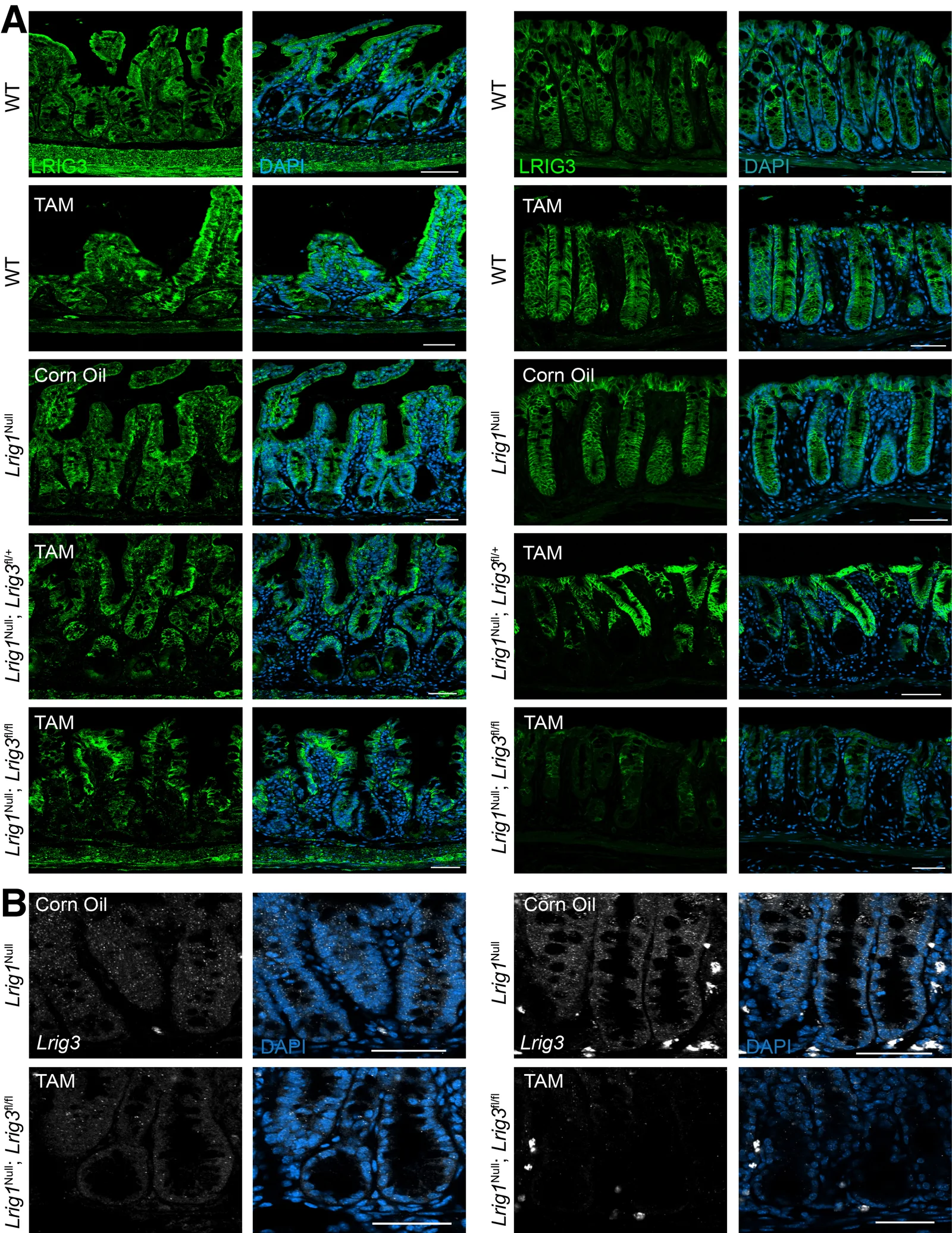
**Inducible deletion of *Lrig3* in *Lrig1*^Null^ ISCs.** (*A*) Analysis of LRIG3 protein by IF in small intestine (SI), *left panel*, and colon, *right panel*, of WT mice, WT mice treated with tamoxifen (TAM), *Lrig1*^Null^ mice treated with corn oil, and *Lrig1*^Null^; *Lrig3*^fl/+^ and *Lrig1*^Null^; *Lrig3*_fl/fl_ mice treated with TAM. LRIG3 IF merged with DAPI (*last column, left and right panel*, respectively). TAM (2 mg) was administered i.p. for 3 consecutive days. TAM and corn oil–treated mice were all sacrificed 7 days after the last TAM dose, with the exception of *Lrig1*^Null^; *Lrig3*^fl/fl^ mice, which were sacrificed 4 days after the last TAM dose. Scale bar, 50 μm. (*B*) Image depicting *Lrig3* RNA by HCR-FISH. *Left panel* represents the SI of mice treated with corn oil (*top row*) or TAM (*bottom row*). *Right panel* represents the colons of mice treated with corn oil (*top row*) or TAM (*bottom row*). *Last row of left and right panel* is LRIG1 HCR-FISH merged with DAPI. TAM (2 mg) treatment was as described above with mice being sacrificed and analyzed 4 days after the last dose of TAM. Scale bar, 50 μm.

**Figure 3:**
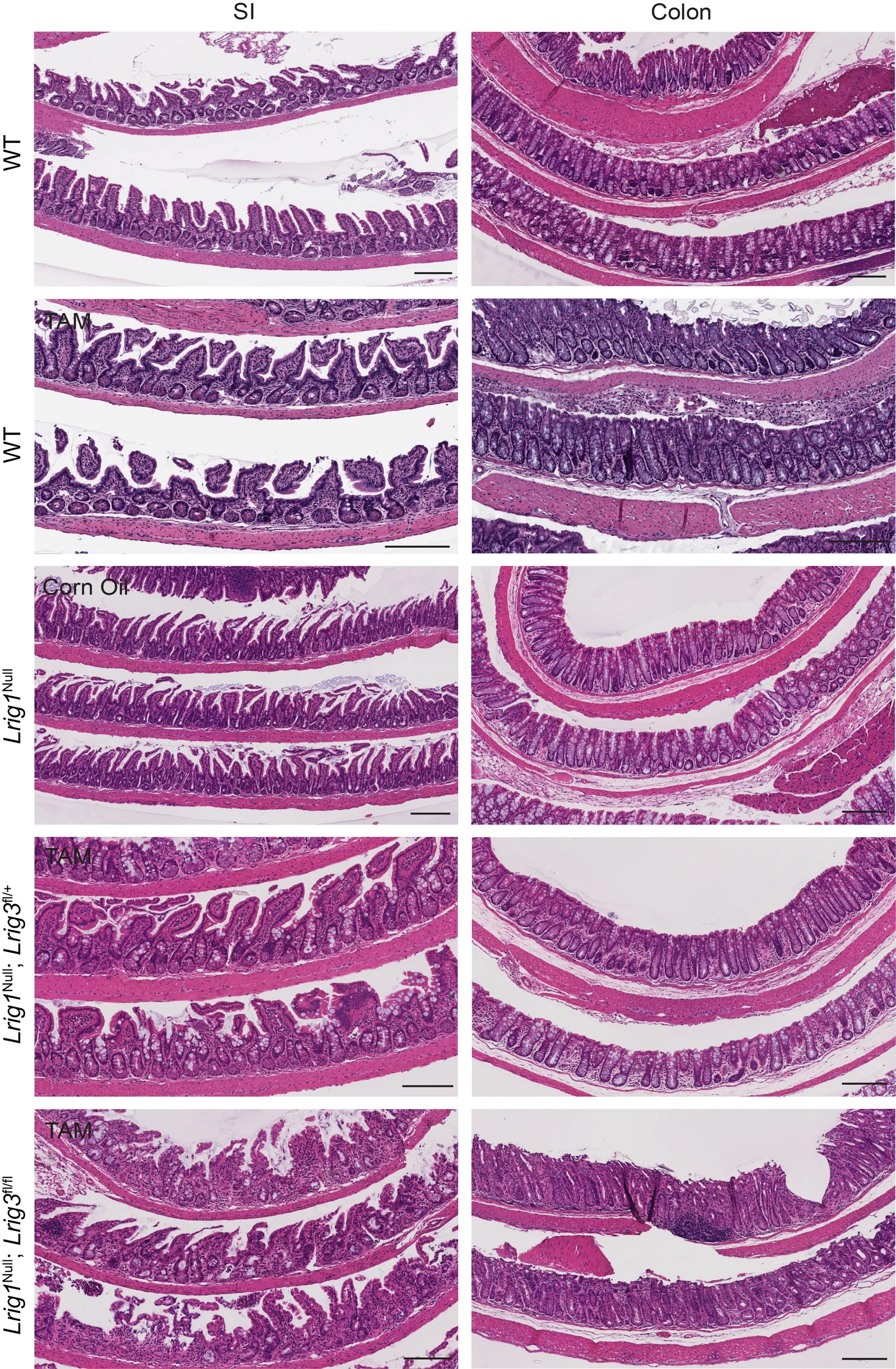
**Inducible deletion of *Lrig3* in *Lrig1*^Null^ cells causes crypt loss and widespread disruption of crypt structure.** Representative, lower magnification images of H&E staining of small intestine (SI) and colonic epithelium from WT mice, WT treated with tamoxifen (TAM), corn oil–treated *Lrig1*^Null^ mice and TAM-treated *Lrig1*^Null^; *Lrig3*^fl/+^ and *Lrig1*^Null^; *Lrig3*^fl/fl^ mice. Mice were treated with TAM and sacrificed for analysis as described in Figure 2. Scale bar, 200 μm.

**Figure 4:**
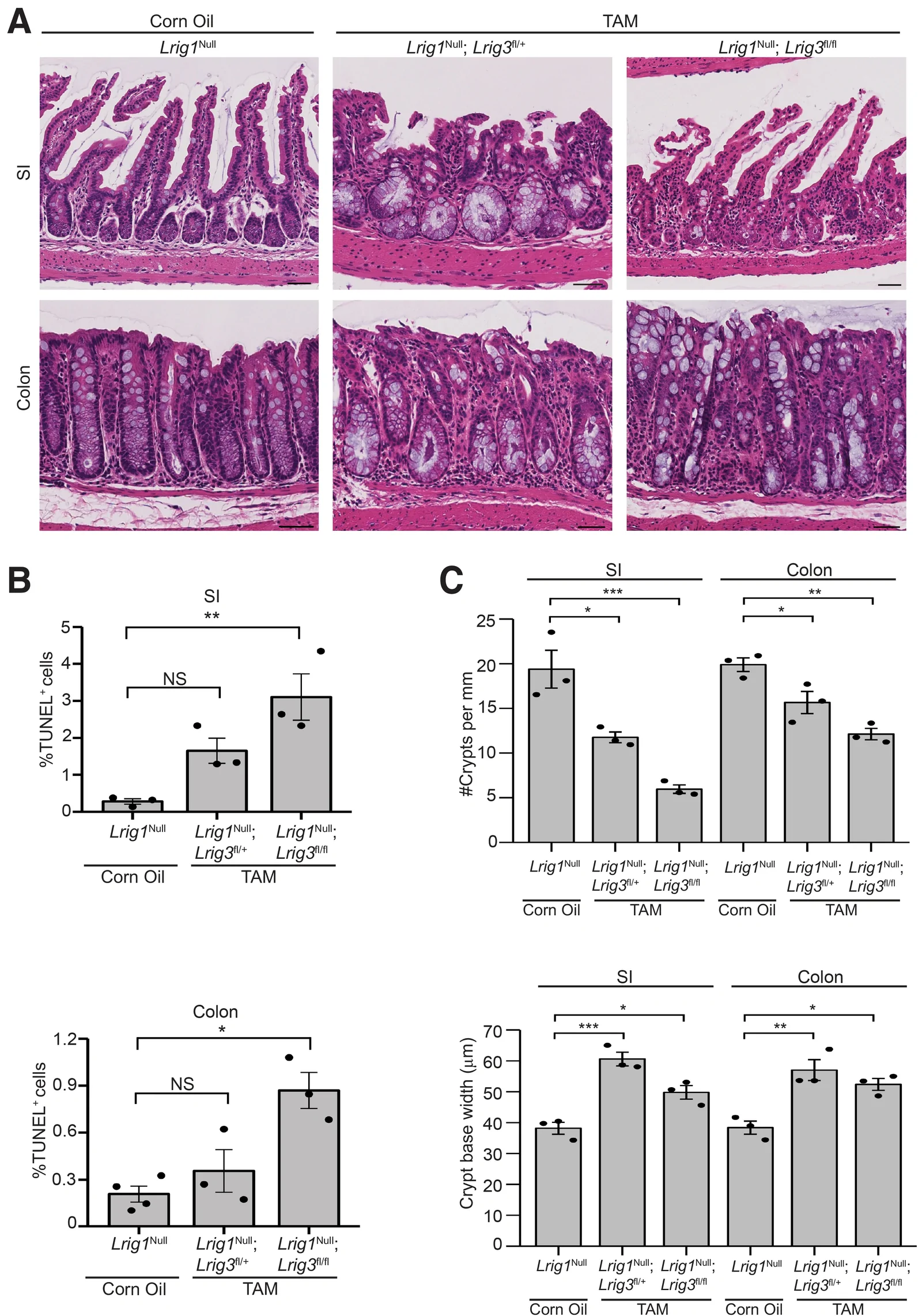
**Inducible deletion of *Lrig3* in *Lrig1*^Null^ cells disrupts crypt homeostasis and causes widespread crypt death.** (*A*). H&E staining of small intestine (SI) and colonic epithelium from corn oil–treated *Lrig1*^Null^ mice and tamoxifen (TAM)-treated *Lrig1*^Null^; *Lrig3*^fl/+^ and *Lrig1*^Null^; *Lrig3*^fl/fl^ mice. Scale bar, 50 μm. (*B*) Quantification of the percentage of TUNEL+ cells in SI (*top panel*) and colon (*bottom panel*) of corn oil–treated *Lrig1*^Null^ mice and TAM-treated *Lrig1*^Null^; *Lrig3*^fl/+^ and *Lrig1*^Null^; *Lrig3*^fl/fl^ mice. (*C*) Number of crypts present in the epithelium of SI and colon (*top panel*) and the width of crypt bases (*lower panel*) of mice described in (*B*). Quantifications were made using N = 3 mice for each of the conditions. Mice were given 3 daily consecutive doses (2 mg) of TAM or corn oil and analyzed 7 days (*Lrig1*^Null^ and *Lrig1*^Null^; *Lrig3*^fl/+^ mice) and 4 days (*Lrig1*^Null^; *Lrig3*^fl/fl^ mice) after the last dose of TAM. Error bars represent the standard error of the mean. NS, no significance. ∗*P* < .05; ∗∗*P* < .01; ∗∗∗*P* < .001 by analysis of variance with a Tukey honestly significant difference (HSD) post hoc test (*B*, *upper graph*), Kruskal–Wallis test with a Dunn post hoc test (*B*, *lower graph*), and analysis of variance with a Tukey HSD post hoc test (*C*).

**Figure 5:**
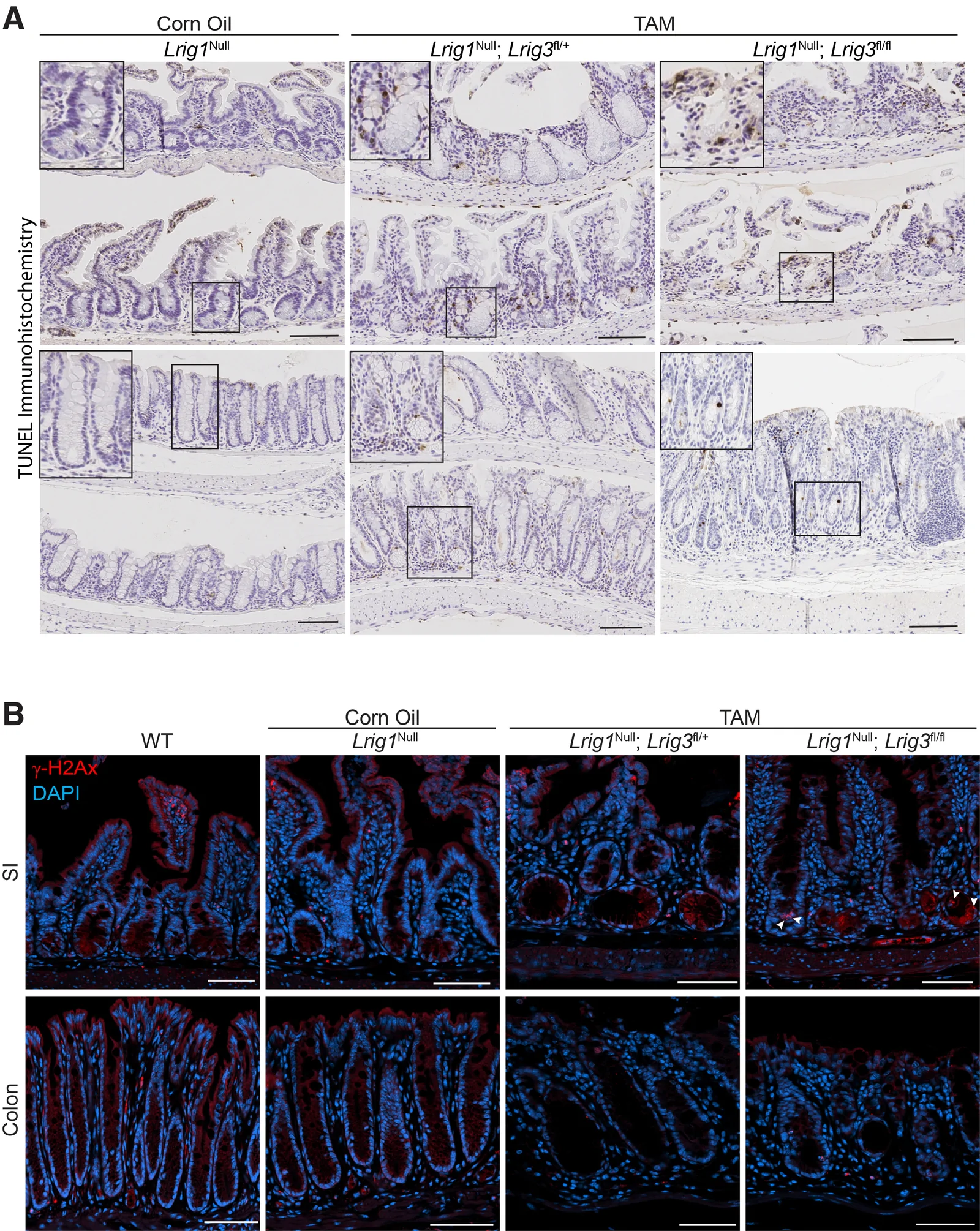
**Loss of LRIG3 in *Lrig1*^Null^ cells results in increased cell death in intestinal crypts.** (*A*) Representative images depicting TUNEL immunohistochemical staining of small intestine (SI; *top row*) and colonic epithelium (*bottom row*) from corn oil–treated *Lrig1*^Null^ mice and tamoxifen (TAM)-treated *Lrig1*^Null^; *Lrig3*^fl/+^ and *Lrig1*^Null^; *Lrig3*^fl/fl^ mice. Corresponding quantification is shown in Figure 4*B*. Scale bar, 200 μm. (*B*) Representative IF image of γ-H2Ax–positive cells in WT mice, corn oil–treated *Lrig1*^Null^ mice and TAM-treated *Lrig1*^Null^; *Lrig3*^fl/+^ and *Lrig1*^Null^; *Lrig3*^fl/fl^ mice. Mice were treated and sacrificed for analysis as described in Figure 2. Scale bar, 50 μm.

Extensive crypt loss and increased cell death after homozygous *Lrig3* deletion in *Lrig1*^Null^ cells complicated any interpretation of LRIG3’s role apart from LRIG1. To minimize distress and generate a milder, more interpretable phenotype, we deleted a single *Lrig3* allele in *Lrig1*^Null^ cells, allowing us to extend the analysis to 7 days after the final dose of tamoxifen rather than the 4-day timepoint in the *Lrig1*^Null^; *Lrig3*^fl/fl^ mice. Similar to the homozygous deletion of *Lrig3*, we observed a significant decrease in *Lrig3* expression at the base of crypts from tamoxifen-treated *Lrig1*^Null^; *Lrig3*^fl/+^ mice relative to corn oil–treated *Lrig1*^Null^ mice (Figure 2*A*). In contrast to tamoxifen-treated *Lrig1*^Null^; *Lrig3*^fl/fl^ mice, the intestinal epithelium from tamoxifen-treated *Lrig1*^Null^; *Lrig3*^fl/+^ mice showed only a modest decrease in crypt number with a much smaller increase in TUNEL positivity compared with their *Lrig1*^Null^; *Lrig3*^fl/fl^ counterparts (Figures 4*B* and *C* and 5*A*). A notable finding in both tamoxifen-treated *Lrig1*^Null^ Lrig3^fl/+^ and *Lrig1*^Null^; Lrig3^fl/fl^ mice was the widening of the crypt base in both the small intestine and colon in comparison to *Lrig1*^Null^ controls. Quantification at the crypt base in corn oil–treated controls showed a width of 38.2 ± 1.9 μm in the small intestine and 38.4 ± 2.1 μm in the colon (Figure 4*C*). The homozygous deletion of *Lrig3* in *Lrig1*^Null^ ISCs increased the crypt width approximately 23% in the small intestine to 49.8 ± 2.2 μm and 27% to 52.4 ± 1.9 μm in the colon (Figure 4*C*). However, widening at the crypt base was more pronounced in tamoxifen-treated *Lrig1*^Null^; *Lrig3*^fl/+^ mice relative to their *Lrig3*^fl/fl^ counterparts (Figure 4*A*). In these samples, the single allele loss of *Lrig3* in *Lrig1*^Null^ ISCs increased the crypt base width ∼37% to 60.5 ± 2.1 μm in the small intestine and ∼33% to 57 ± 3.4 μm in the colon, relative to *Lrig1*^Null^ controls (Figure 4*C*). The increased cell death and crypt loss in tamoxifen-treated *Lrig1*^Null^; *Lrig3*^fl/fl^ mice is the likely explanation for the diminished effect on the crypt width relative to tamoxifen-treated *Lrig1*^Null^; Lrig3^fl/+^ mice (Figure 4*C*). Moreover, the cells crowding the small intestinal crypt base in both *Lrig3* heterozygous and homozygous tamoxifen-treated mice contained what appeared to be lysozyme granules, suggesting precocious differentiation to a secretory or Paneth cell lineage (Figures 3 and 4*A*). Although colonic crypts do not typically have lysozyme-positive Paneth cells, the widened crypts present in tamoxifen-treated colonic tissue were heavily populated by mucin-positive secretory cells, consistent with a secretory cell and/or goblet cell hyperplasia (Figure 4*A*). Together, these data indicate that in the absence of *Lrig1*, the loss of LRIG3 in *Lrig1*-expressing cells triggers aberrant secretory cell differentiation. No overt phenotype was observed with the heterozygous or homozygous loss of *Lrig3* in the *Lrig1*^CreERT2/+^ heterozygous background. These results underscore the necessity of LRIG1 loss to uncover *Lrig3* heterozygous and null phenotypes within *Lrig1*-expressing cells.

### Inducible *Lrig3* Deletion Shifts Stem Cells Toward Immature Paneth/Goblet Intermediate Cells

To determine if widening of the crypt base was due to Paneth cell hyperplasia, we examined lysozyme IF in the small intestine of tamoxifen-treated *Lrig1*^Null^; *Lrig3*^fl/+^ and *Lrig1*^Null^; *Lrig3*^fl/fl^ mice. A substantial increase in lysozyme-positive cells at the small intestinal crypt base of tamoxifen-treated mice was observed, relative to WT and corn oil–treated controls (Figures 6*A* and 7*A*). Because Paneth and goblet cells are closely related and can emerge from a common precursor,^33^^,^^34^ we investigated whether deletion of *Lrig3* in *Lrig1*^Null^ ISCs influences goblet cell differentiation. The number of MUC2^+^ cells, a common goblet cell marker, increased in tamoxifen-treated *Lrig1*^Null^; *Lrig3*^fl/+^ and *Lrig1*^Null^; *Lrig3*^fl/fl^ small intestinal crypts in comparison to controls (Figure 6*A*). Of interest, there appeared to be a strong overlap in the staining pattern of MUC2 and lysozyme in the expanded crypt bases of *Lrig3*-deficient *Lrig1*^Null^ mice, suggesting coexpression in the same cell (Figure 6*A*). Indeed, lysozyme-positive cells located at the base of the expanded crypts were observed to coexpress trefoil factor 3, a goblet cell marker (Figure 6*B*). Once again, the much less dramatic appearance of this phenotype in tamoxifen-treated *Lrig1*^Null^; *Lrig3*^fl/fl^ mice relative to tamoxifen-treated *Lrig1*^Null^; *Lrig3*^fl/+^ mice (Figures 6*A* and *B* and 7*A*) is likely due to the increased cell death and crypt loss resulting from *Lrig1* and *Lrig3* homozygous deletion (Figure 4). Secretory cells in the small intestine that have characteristics of both goblet and Paneth cells are a rare population often referred to as “intermediate cells.”^35^ Transmission electron microscopy (TEM) revealed that the MUC2 and lysozyme double-positive cells at the crypt base in Lrig3-deficient *Lrig1*^Null^ mice contained mucin-containing secretory granules with electron-dense lysozyme cores, a defining feature of intermediate cells (Figure 6*C*). Moreover, several fusion events between these aberrant mucin and lysozyme granules and what appears to be more normal-looking lysozyme granules were observed (Figure 6*C*). Thus, in the small intestine, we provide compelling evidence that intermediate cells explain the widened crypts at the base of *Lrig1*-expressing cells that are *Lrig1*^Null^ and have either heterozygous or homozygous loss of *Lrig3*.

**Figure 6:**
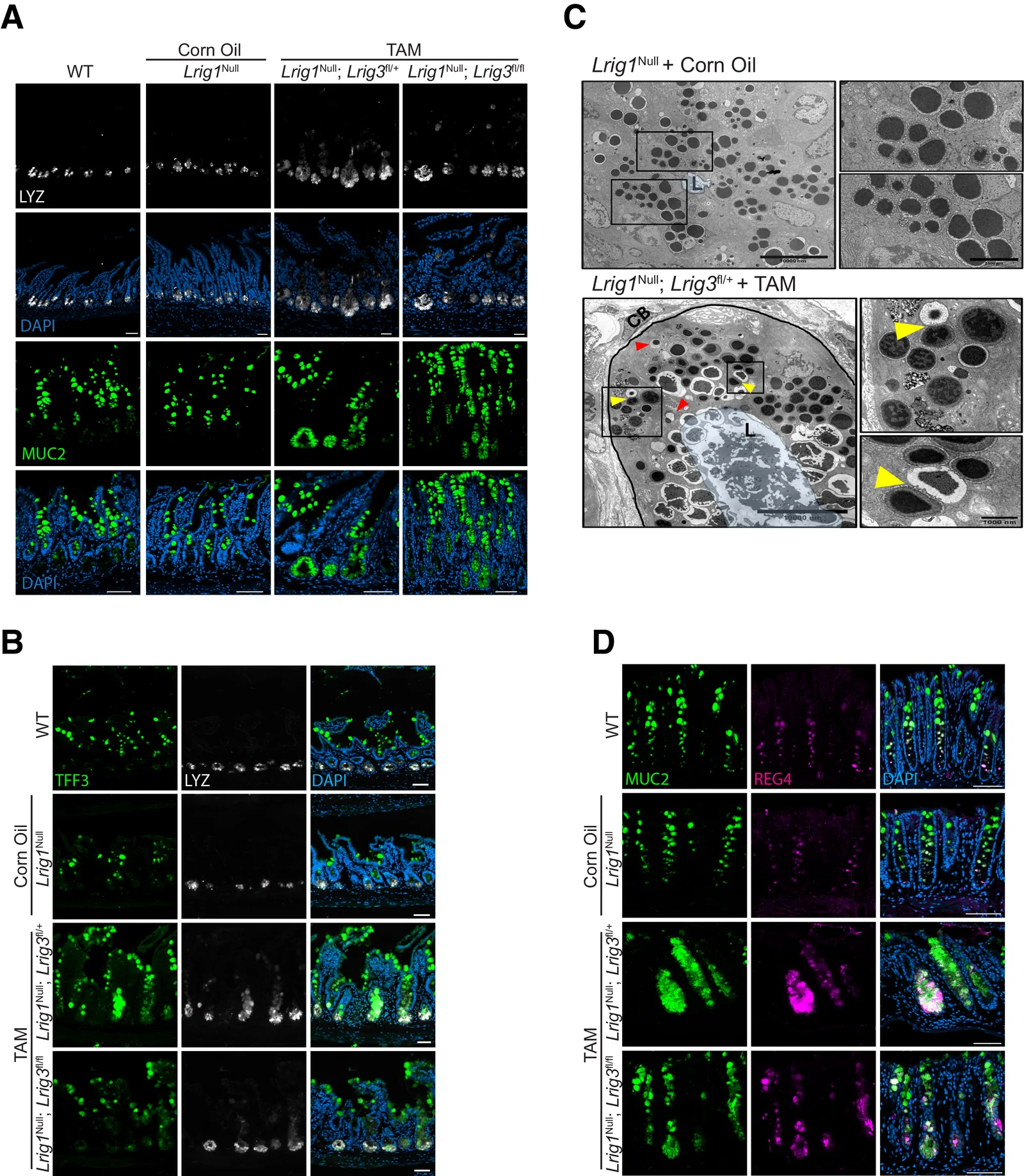
**LRIG3 loss in *Lrig1*^Null^ cells leads to expansion of the crypt base due to overproduction of LYZ^+^/MUC2^+^ immature secretory cells.** Representative IF of small intestine (SI) tissue (*A* and *B*) and colonic tissue (*D*) from untreated WT, corn oil–treated *Lrig1*^Null^, and tamoxifen (TAM)-treated *Lrig1*^Null^; *Lrig3*^fl/+^ and *Lrig1*^Null^; *Lrig3*^fl/fl^ mice stained for (*A*) lysozyme and MUC2, (*B*) lysozyme and trefoil factor 3 (TFF3), or (*D*) REG4 and MUC2 with images merged with DAPI staining. The timepoint of IF is as described in Figure 2. Scale bar, 50 μm. (*C*) TEM images of SI crypts from corn oil–treated *Lrig1*^Null^ mice (*top*; bottom-up view of crypt) and TAM-treated *Lrig1*^Null^; *Lrig3*^fl/+^ mice (*bottom*, cross-sectional view) approximately 3 days after final TAM treatment. CB, crypt bottom, L, lumen. *Red arrowheads* indicate the presence of lysozyme- and mucin-containing secretory granules. *Yellow arrowheads* indicate fusion between lysozyme and mucin double-positive granules and other lysozyme granules. *Black boxes* indicate magnified areas.

**Figure 7:**
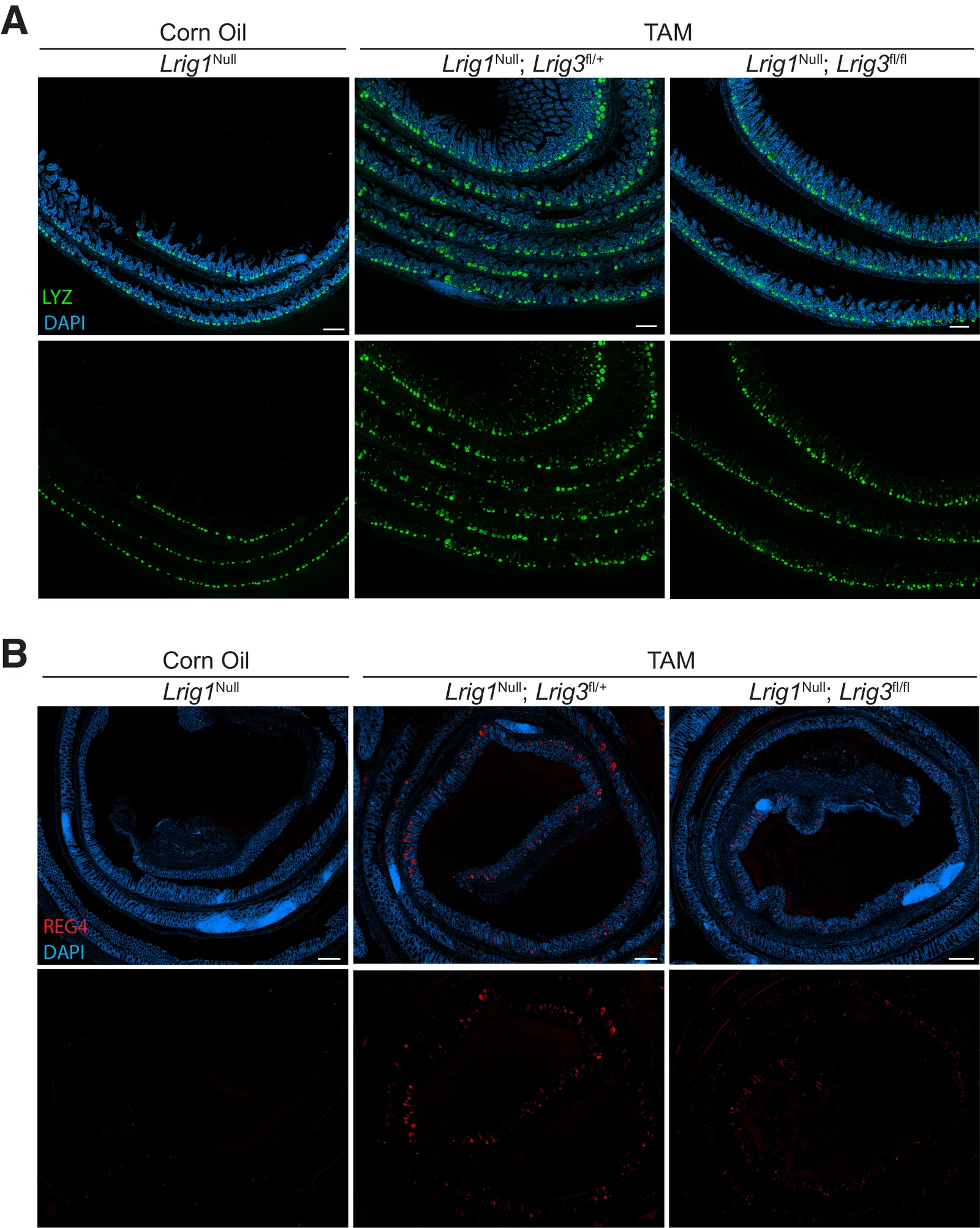
**LRIG3 loss in *Lrig1*^Null^ cells leads to increased lysozyme (LYZ) and REG4 in the small intestine and colon, respectively.** Representative, low magnification, IF images of small intestine (SI) (*A*) and colonic tissue (*B*) from corn oil–treated *Lrig1*^Null^ mice and tamoxifen (TAM)-treated *Lrig1*^Null^; *Lrig3*^fl/+^ and *Lrig1*^Null^; *Lrig3*^fl/fl^ mice stained for (*A*) lysozyme. IF analysis was performed as described in Figure 2. Scale bar, 200 μm or (*B*) REG4. Scale bar, 400 μm.

Although murine colonic crypts typically lack Paneth cells, they do have REG4^+^ Paneth-like cells called DSCs.^36^ Tamoxifen-treated *Lrig1*^Null^; *Lrig3*^fl/+^ and *Lrig1*^Null^; *Lrig3*^fl/fl^ colonic crypts showed a marked increase in REG4^+^ DSCs coexpressing the goblet marker MUC2 (Figures 6*D* and 7B). As noted above, the increased cell death and overall crypt loss in tamoxifen-treated *Lrig1*^Null^; *Lrig3*^fl/fl^ mice likely explain the less-dramatic phenotype related to the increased presence of REG4, MUC2 double-positive DSCs. To clarify whether the increase in Paneth/goblet intermediate cells and DSCs was due to cell division–driven differentiation or a direct transition from a stem cell state to an immature secretory cell fate, we injected tamoxifen-treated *Lrig1*^Null^; *Lrig3*^fl/+^ and *Lrig1*^Null^; *Lrig3*^fl/fl^ mice with the thymidine analogue 5-ethynyl-2'-deoxyuridine (EdU) 4 hours prior to dissection, either 7 days (*Lrig1*^Null^; *Lrig3*^fl/+^) or 4 days (*Lrig1*^Null^; *Lrig3*^fl/fl^) after the final tamoxifen injection. Analysis of EdU incorporation showed that the majority of EdU^+^ cells in corn oil–treated *Lrig1*^Null^ mice localized to the highly proliferative transit-amplifying zone, with sporadic EdU^+^ cells at the crypt base (Figure 8*A–C*). However, in tamoxifen-treated mice, we observed a highly disorganized staining pattern, with many expanded crypt bottoms devoid of EdU^+^ cells (Figure 8*A–C*). Given the lack of proliferation, these findings support a model in which the loss of *Lrig3* in *Lrig1*^Null^ ISCs causes a shift in ISC identity to a more differentiated postmitotic state that resembles Paneth/goblet intermediate cells in the small intestine and DSCs in the colon.

**Figure 8:**
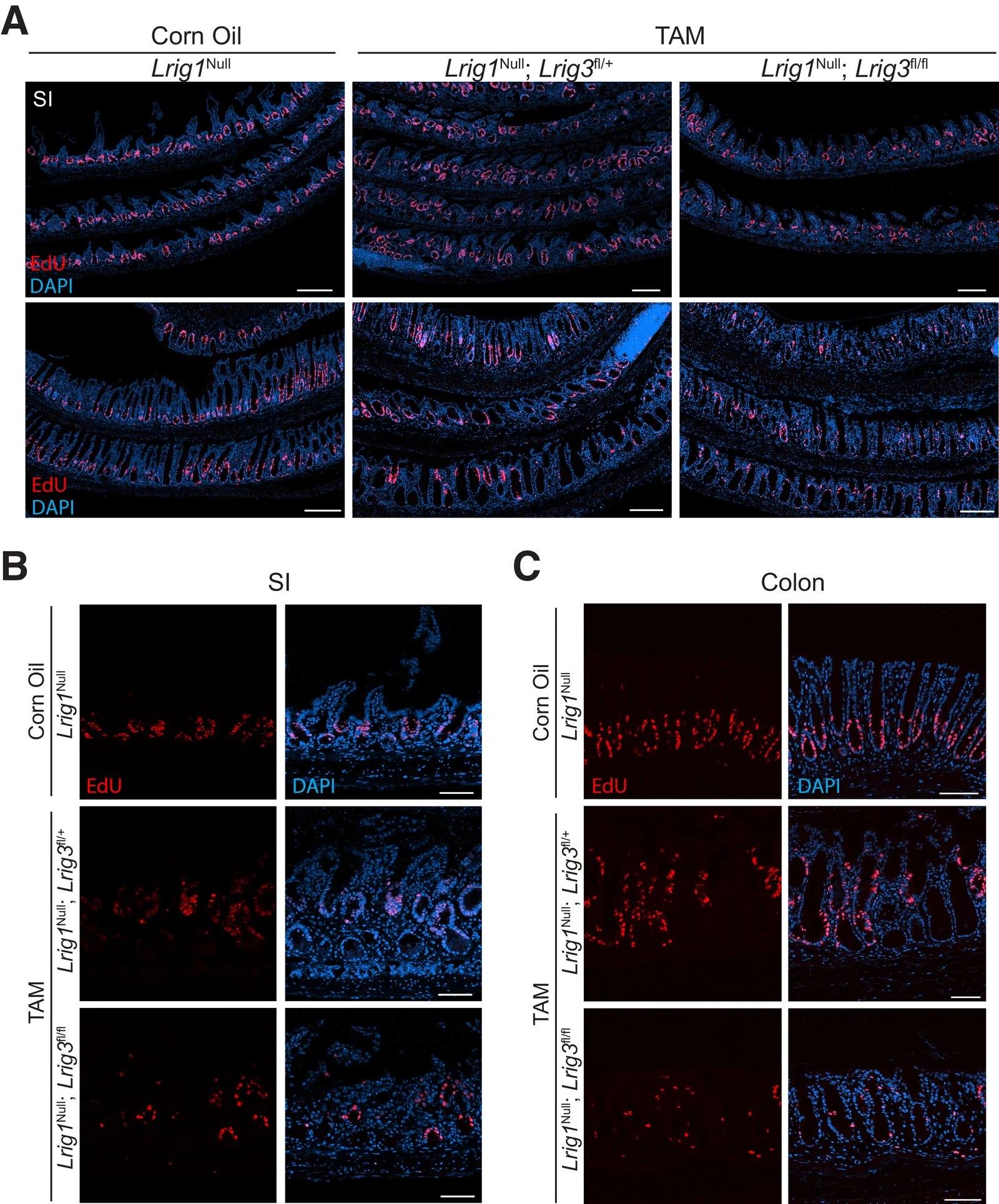
**Crypt expansion caused by the deletion of *Lrig3* is not due to proliferation.** (*A*) IF showing EdU (injected with a single EdU dose 4 hours prior to dissection) incorporation over a large area of small intestine (SI; *top row*) and colonic (*bottom row*) tissue from corn oil–treated *Lrig1*^Null^ mice and tamoxifen (TAM)-treated *Lrig1*^Null^; *Lrig3*^fl/+^ and *Lrig1*^Null^; *Lrig3*^fl/fl^ mice. Scale bar, 200 μm. Representative IF showing EdU incorporation in (*B*) small intestinal (SI) and (*C*) colonic tissue from corn oil–treated *Lrig1*^Null^ mice and TAM-treated *Lrig1*^Null^; *Lrig3*^fl/+^ and *Lrig1*^Null^; *Lrig3*^fl/fl^ mice, injected with EdU as in (*A*). Scale bar, 50 μm. Analysis was performed as described in Figure 2. Scale bar, 50 μm.

### LRIG3 Loss in *Lrig1*^Null^ Cells Results in a Loss of Stem Cell Identity and Emergence of an Immature Paneth/Goblet Cell Lineage

To gain an understanding of early changes in gene expression occurring in intestinal crypts following the deletion of *Lrig3* in *Lrig1*^Null^ ISCs, we performed scRNA-seq analysis on small intestinal and colonic crypts isolated from corn oil–treated *Lrig1*^Null^, tamoxifen-treated *Lrig1*^Null^; *Lrig3*^fl/+^, and tamoxifen-treated *Lrig1*^Null^; *Lrig3*^fl/fl^ mice 3 days after the last tamoxifen injection. Supporting our IF data, we observed an increased expression of secretory lineage markers (*Atoh1*, *Dll1*, *Dll4*), Paneth cell markers (*Lyz1*, *Mmp7*), and goblet cell markers (*Muc2*, *Tff3*, *Spdef*, *Klf4*) with the loss of 1 *Lrig3* allele; this pattern of expression was further increased with the loss of the second allele (Figures 9*A* and 10*A*). Uniform Manifold Approximation and Projection (UMAP) analysis revealed that the cell cluster in tamoxifen-treated small intestinal samples, corresponding to the goblet cell cluster in corn oil–treated controls, showed an elevated expression of *Muc2* as well as the Paneth cell markers, *Lyz* and *Mmp7*, prompting us to relabel this cluster to reflect an intermediate Paneth/goblet cell state (Figure 9*B* and *C*). Cell-type compositional analysis showed a distinct increase in the proportion of these Paneth/goblet intermediate cells in the small intestinal crypts from tamoxifen-treated mice that was accompanied by a marked decrease in the proportion of stem cells (Figure 9*B*). Analysis of colonic crypts from tamoxifen-treated mice revealed a higher proportion of goblet cells (Figure 10*B* and *C*) with elevated expression of the DSC marker *Reg4* in conjunction with *Muc2* (Figure 10*C*). A decrease in the proportion of stem cells in colonic crypts was observed following the loss of *Lrig3* in *Lrig1*^Null^ ISCs (Figure 10*B* and *C*). Of note, there was also elevated expression of the Paneth cell markers *Lyz1* and *Mmp7* (Figure 10*C*).

**Figure 9:**
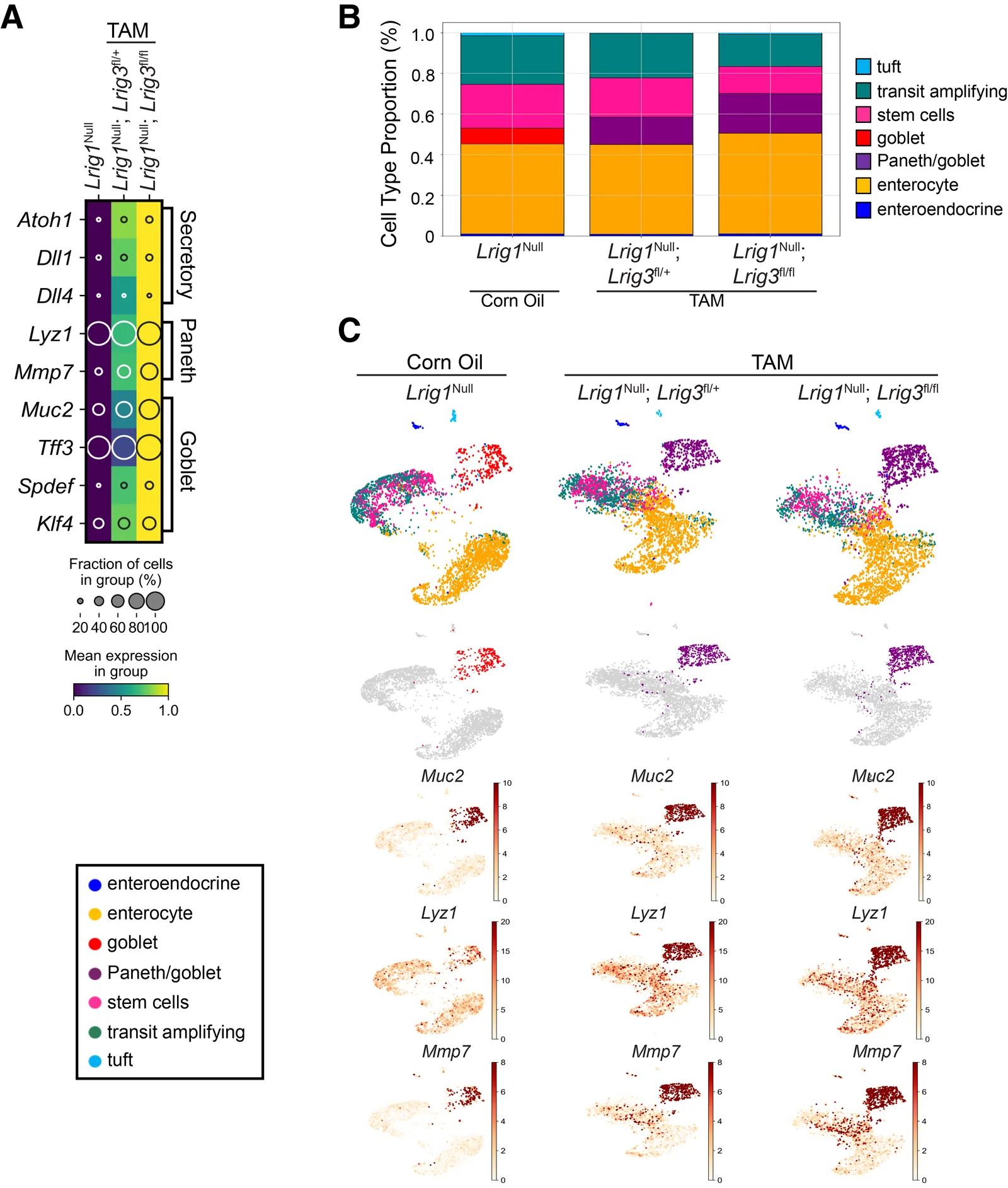
**scRNA-seq analysis shows that LRIG3 loss in *Lrig1*^Null^ cells results in the loss of stem cell identity and the emergence of an immature Paneth**/**gobletintermediatecell lineage.** (*A*) Bubble heatmap plot showing scRNA-seq gene expression of markers used to identify the indicated cell state. (*B*) Analysis showing the proportion of the indicated cell types present in corn oil–treated *Lrig1*^Null^, and tamoxifen (TAM)-treated *Lrig1*^Null^; *Lrig3*^fl/+^ and *Lrig1*^Null^; *Lrig3*^fl/fl^ samples. (*C*) UMAP analysis indicating cell-type assignments for clusters generated based on canonical mouse gene markers, with the goblet and the Paneth/goblet cell population clusters highlighted in *red and purple*, respectively. The *3 bottom rows* show UMAP plots colored by gene expression levels of *Muc2*, *Lyz1*, and *Mmp7*. scRNA-seq analysis for each condition was performed on crypts isolated approximately 3 days after the last TAM treatment (N = 2 mice per condition).

**Figure 10:**
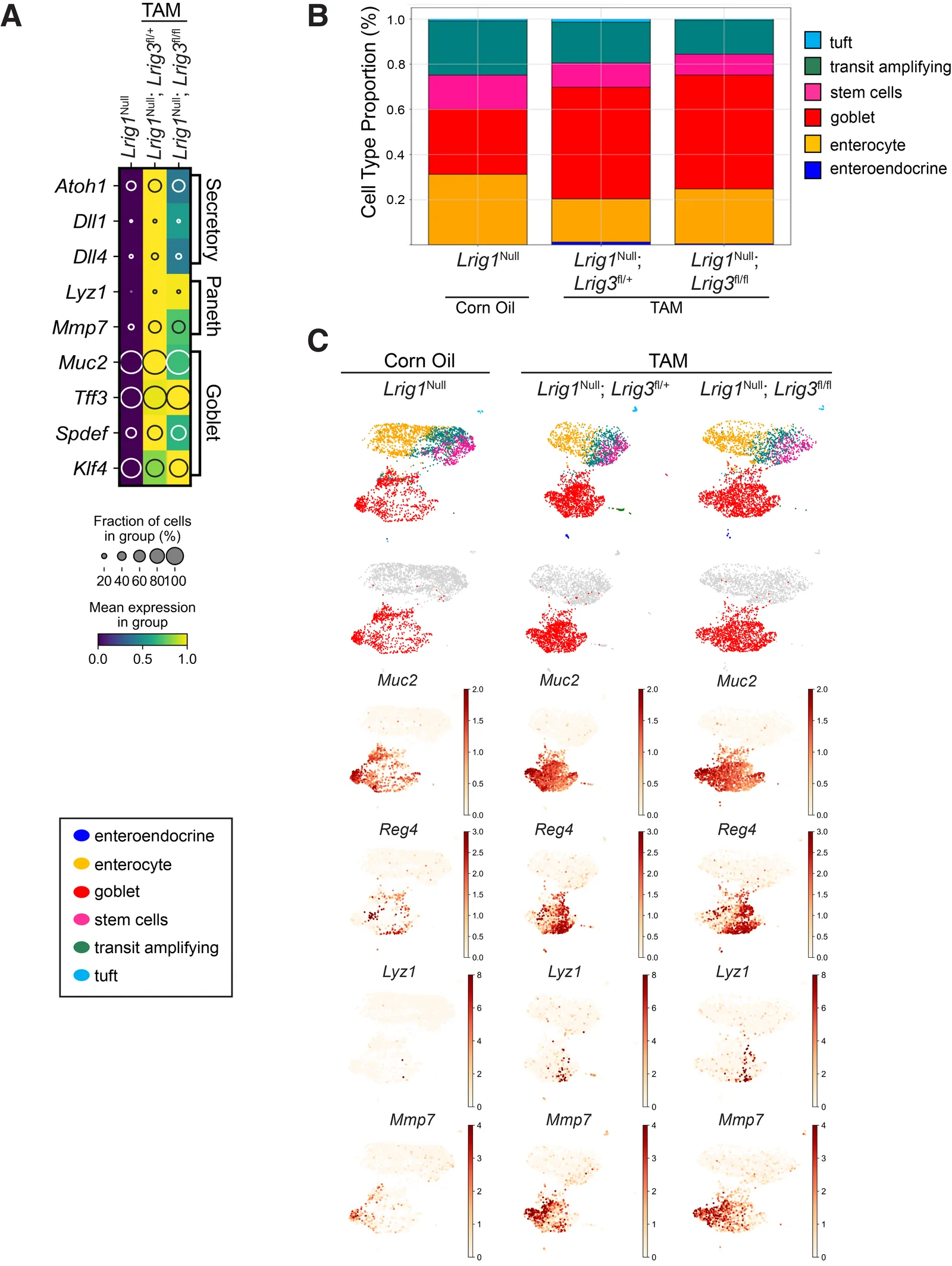
**scRNA-seq analysis shows the deletion of *Lrig3* in *Lrig1*^Null^ cells resulted in the expansion of deep secretory cells at expense of stem cells in colonic crypts.** (*A*) Bubble heatmap plot showing the scRNA-seq gene expression of markers used to identify the indicated cell state. (*B*) Analysis showing the proportions of cell types present in corn oil–treated *Lrig1*^Null^ mice and tamoxifen (TAM)-treated *Lrig1*^Null^; *Lrig3*^fl/+^ and *Lrig1*^Null^; *Lrig3*^fl/fl^ mice. (*C*) UMAP plot showing cell-type assignments for clusters generated based on canonical mouse gene markers. (*C*) UMAP plot with the goblet cell population clusters in *red* (*top row*). The next *4 rows* show UMAP plots colored by gene expression levels of *Muc2* (goblet cell marker), *Reg4* (deep secretory cell marker), *Lyz1* (Paneth cell marker), and *Mmp7* (Paneth cell marker). scRNA-seq analysis for each condition was performed on crypts isolated approximately 3 days after final treatment (N = 2 mice per condition).

The increased cell death, crypt loss, and Paneth/goblet intermediate cell hyperplasia following the heterozygous and homozygous deletion of *Lrig3* in *Lrig1*^Null^ cells suggest a dysfunction in homeostatic maintenance and differentiation of ISCs. An analysis of gene expression changes showed the decreased expression of multiple ISC markers (*Lgr5*, *Olfm4*, *Smoc2*) in tamoxifen-treated small intestinal crypts (Figure 11*A*). In contrast, the colons of tamoxifen-treated mice showed a marked decrease in the expression of *Smoc2*, with a decrease in *Lgr5* expression occurring only in tamoxifen-treated *Lrig1*^Null^; *Lrig3*^fl/fl^ mice (Figure 11*A*). The inducible loss of *Lrig3* also triggered increased expression of *Ascl2*, *Clu*, *Emp1*, and *Tob2* (Figure 11*A*), which are associated with injury-induced regeneration by stem cell populations.^37^, ^38^, ^39^, ^40^, ^41^ The crypts in both the small intestines and the colons of tamoxifen-treated mice also showed an increased expression of cell-cycle exit markers (*Gadd45a*, *Sfn*, *Trp53*, *Cdkn1a*) (Figure 11*A*). In addition, the increased expression of several markers of cellular senescence (*Glb1*, *Lmnb1*, *Gdf15*, *Rab13*) was observed (Figure 11*A*). Indeed, an additional analysis showed an increased expression of β-galactosidase, a well-defined marker of senescence, throughout the crypt epithelium of tamoxifen-treated mice, relative to controls (Figure 11*D* and *E*). In addition to a decrease in the stem cell proportion (Figure 9*A*), UMAP analysis of small intestinal crypts from tamoxifen-treated mice showed a drastic shift in the UMAP coordinate of the stem cell population towards enterocytes, suggesting a more differentiated state (Figure 11*B*). Examination of gene expression in this altered cluster of stem cells revealed a marked decrease in the stem cell score, decreased *Lrig3* expression, loss of *Lgr5*, and increased expression of *Cdkn1a* (Figure 11*B*). In the colon, despite a decrease in the overall number of stem cells (Figure 10*B*), reduced *Lrig3* and *Lgr5* expression, and increased *Cdkn1a* expression, the UMAP clustering and stem cell scores remained largely unchanged (Figure 11*C*). IF showed a dramatic decrease in the expression of both olfactomedin 4 (OLFM4) and SRY-box transcription factor 9 (SOX9) in the small intestinal crypts of tamoxifen-treated *Lrig1*^Null^; *Lrig3*^fl/+^ mice; OLFM4 and SOX9 decreased further in *Lrig1*^Null^; *Lrig3*^fl/fl^ mice (Figure 12*A*). Moreover, immunoblot analysis showed a loss of OLFM4 levels in the small intestine as early as 3 days after the last tamoxifen injection (Figure 14*B*). Although OLFM4 is not expressed in the murine colonic epithelium, a decrease in SOX9^+^ cells at the crypt base of the colon was observed following both heterozygous and homozygous deletion of *Lrig3* in *Lrig1*^Null^ cells (Figure 12*B*). Under normal homeostatic conditions, ISCs transit to highly proliferative transit-amplifying cells, which give rise to more differentiated cell types.^1^ The inducible deletion of *Lrig3* in *Lrig1*^Null^ cells resulted in reduced Ki67 staining throughout the small intestinal and colonic crypts, suggesting that loss of Lrig3 counters the increased proliferation associated with Lrig1 loss^3^ (Figure 12*A* and *B*). We also observed a dramatic decrease in the expression of *Fgfbp1* (Figure 11*A*), which marks a population of proliferative stem cells located in the transit-amplifying zone that migrate upwards and downwards in the crypt.^42^ These data show that the combined loss of *Lrig1* and *Lrig3* in *Lrig1*-expressing ISCs markedly disrupts intestinal crypt homeostasis. In this setting, there appears to be a collapse of the crypt stem cell niche, with stem cells adopting an immature secretory cell fate.

**Figure 11:**
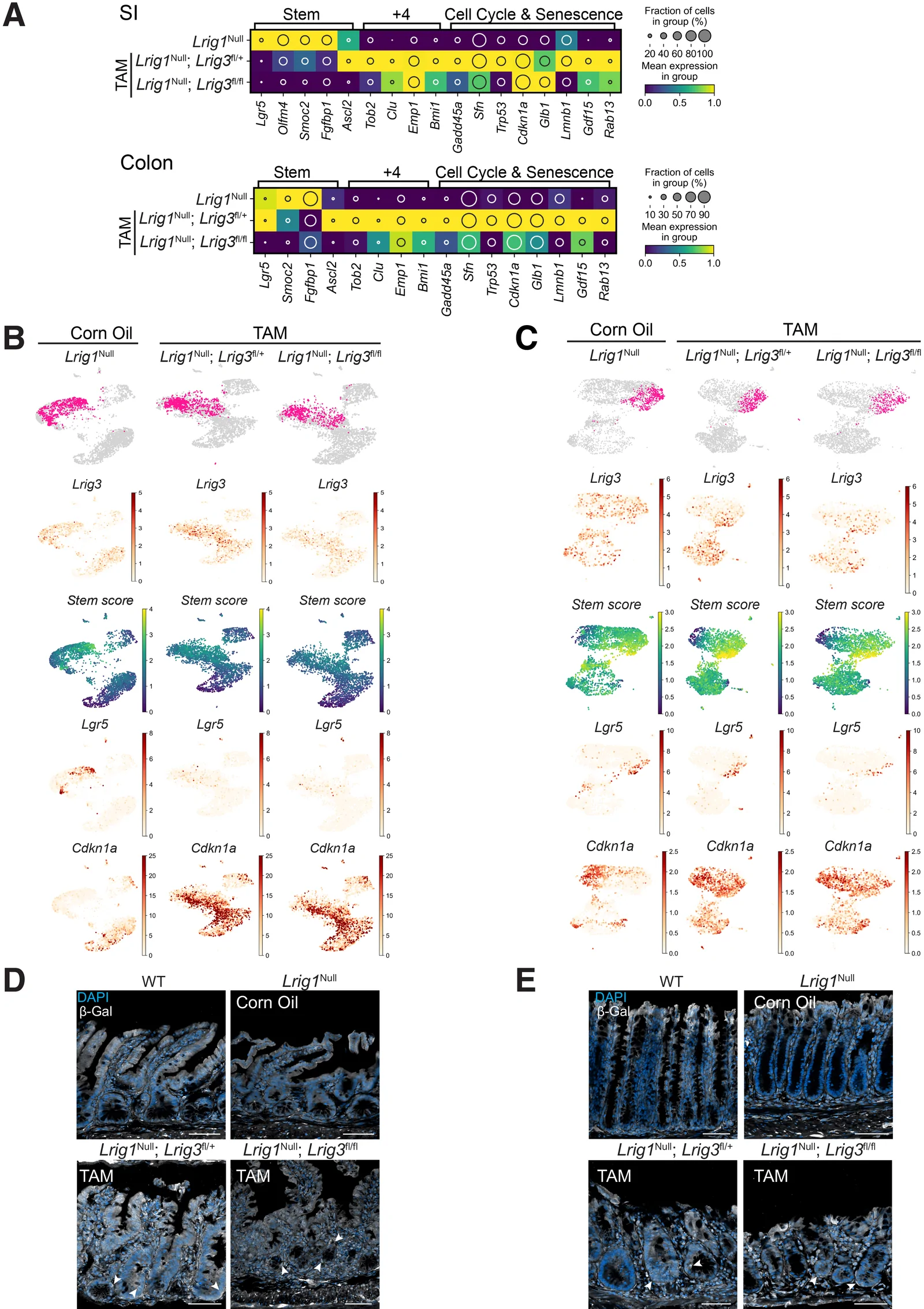
**Tamoxifen-induced deletion of *Lrig3* in *Lrig1*^Null^ cells causes decreased ISC marker expression with a corresponding increase in cell-cycle exit and senescence markers.** (*A*) Bubble heatmap plot showing scRNA-seq gene expression of markers used to identify the indicated cell state or activity. *Upper plot* is small intestine (SI); *lower plot* is colon. UMAP analysis of isolated crypts from corn oil–treated *Lrig1*^Null^ and tamoxifen (TAM)-treated *Lrig1*^Null^; *Lrig3*^fl/+^ and *Lrig1*^Null^; *Lrig3*^fl/fl^ mice from (*B*) SI and (*C*) colon with the stem cell population cluster highlighted in *pink*, and the stem score in the *thirdrow*. The *second row and the last 2 rows* of both (*B*) and (*C*) show UMAP plots colored by gene expression levels of *Lrig3*, *Lgr5*, and *Cdkn1a,* respectively. scRNA-seq analysis for each condition was performed on crypts isolated approximately 3 days after final TAM treatment (N = 2 mice per condition). Representative IF images of (*D*) SI and (*E*) colonic tissue from corn oil–treated *Lrig1*^Null^ mice, and TAM-treated *Lrig1*^Null^; *Lrig3*^fl/+^ and *Lrig1*^Null^; *Lrig3*^fl/fl^ mice stained for β-galactosidase (β-Gal). IF of intestinal tissue was done as described in Figure 2. Scale bar, 50 μm.

**Figure 12:**
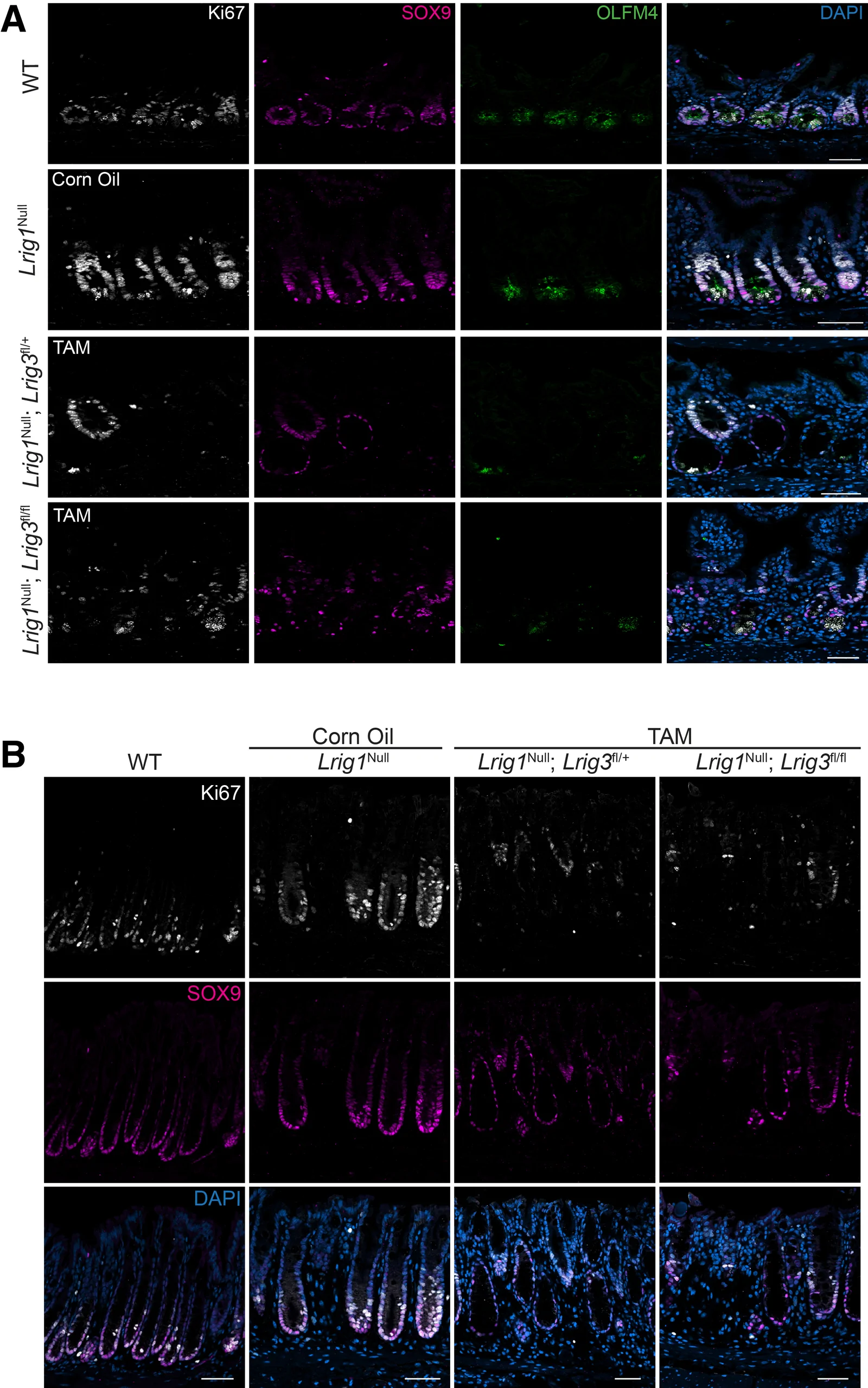
**The loss of LRIG3 in *Lrig1*^Null^ cells results in decreased proliferation and intestinal stem cells in small intestinal (SI) and colonic crypts.** Representative IF images of (*A*) SI and (*B*) colonic tissue from untreated WT mice, corn oil–treated *Lrig1*^Null^ mice, and tamoxifen (TAM)-treated *Lrig1*^Null^; *Lrig3*^fl/+^ and *Lrig1*^Null^; *Lrig3*^fl/fl^ mice stained for Ki67, SOX9, and OLFM4. The *last column* is merged images with DAPI. IF of intestinal tissue was performed as described in Figure 2. Scale bar, 50 μm.

**Figure 13:**
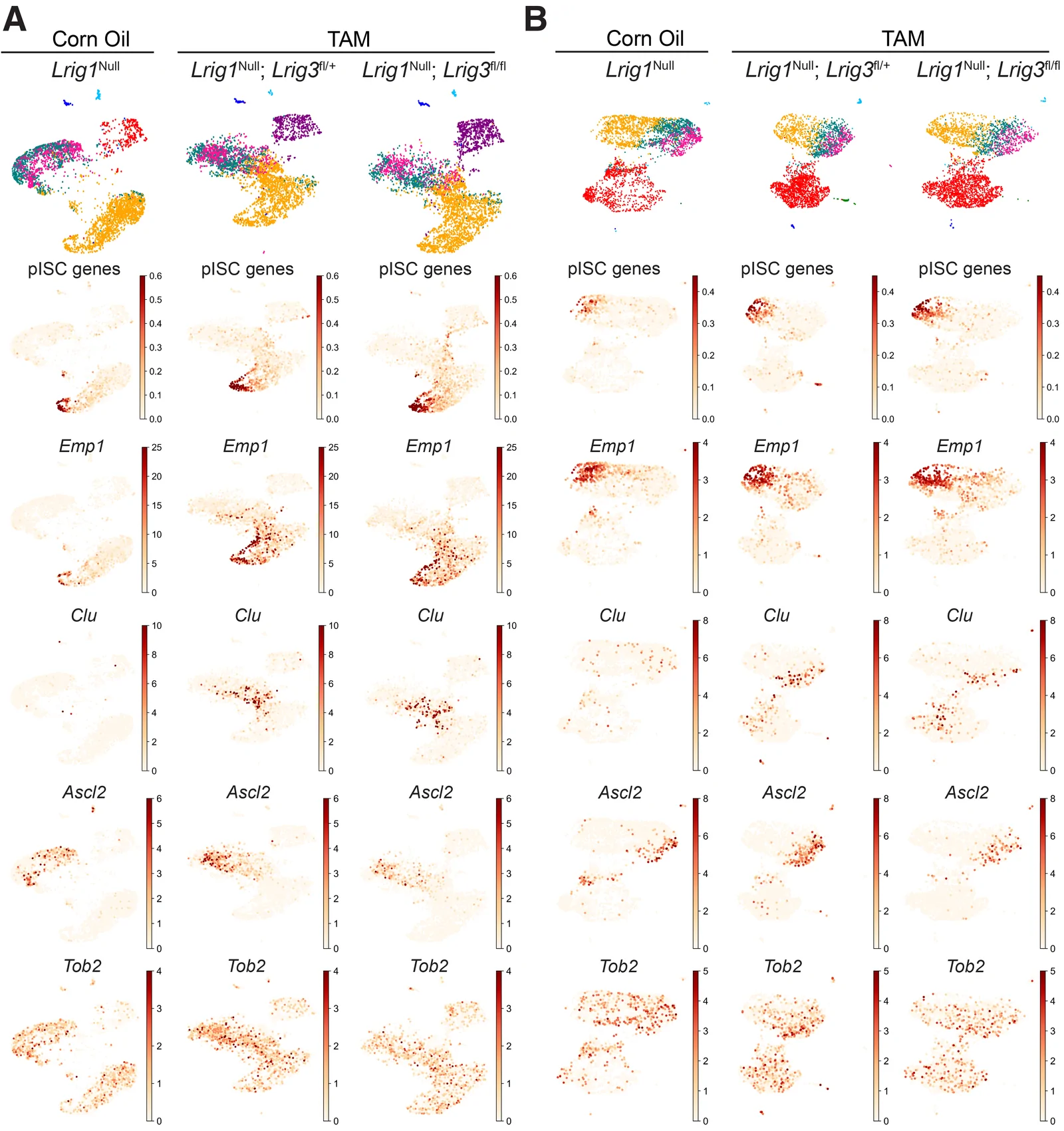
**LRIG3 loss in *Lrig1*^Null^ cells trigger damage response and activation ofrevival stem cellsand pISCs.** UMAP analysis of cell clusters with overlayed gene expression using scRNA-seq data generated from isolated (*A*) small intestinal (SI) and (*B*) colonic crypts. For (*A*) and (*B*), the *first row* is a UMAP plot showing cell-type assignments for clusters generated based on canonical mouse gene markers. The next 5 rows are UMAP plots colored by gene expression levels of pISC genes (group of 31 genes previously shown to define pISC population1), *Emp1* (marker of pISC population), *Clu* (marker of revival stem cells), *Ascl2* (stem cell marker), and *Tob2* (marker of pISC population). scRNA-seq analysis for each condition was performed on crypts isolated approximately 3 days after final treatment (N = 2 mice per condition).

**Figure 14:**
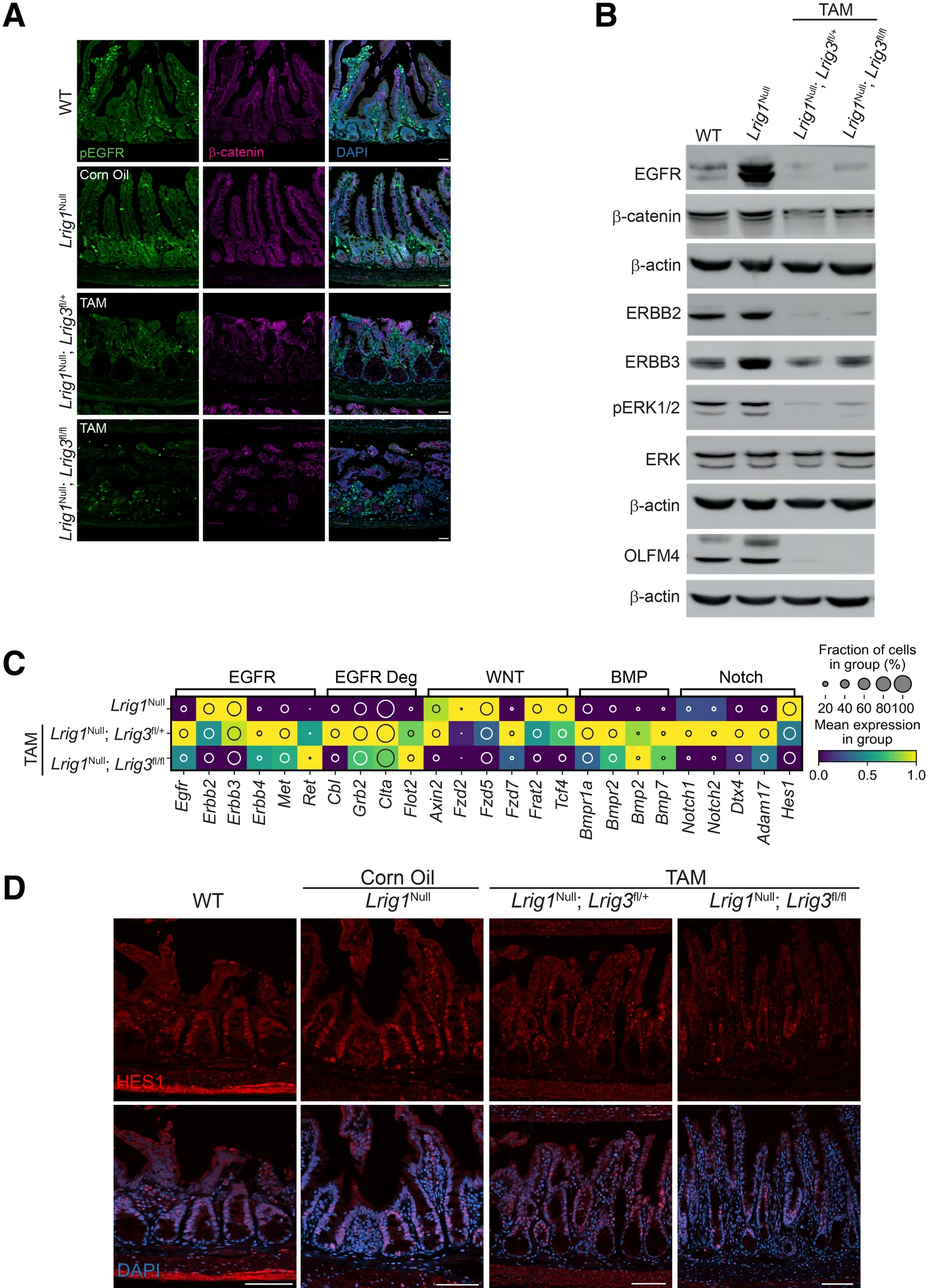
**Tamoxifen-induced deletion of *Lrig3* in *Lrig1*^Null^ cells results in decreased EGFR, WNT, and Notch signaling in small intestine (SI).** (*A*) Representative IF images of SI tissue from untreated WT mice, corn oil–treated *Lrig1*^Null^ mice, and tamoxifen (TAM)-treated *Lrig1*^Null^; *Lrig3*^fl/+^ and *Lrig1*^Null^; *Lrig3*^fl/fl^ mice stained for β-catenin and pEGFR (pY-1068). (*B*) Immunoblotting of the indicated proteins in lysates of SI crypts isolated from WT, corn oil–treated *Lrig1*^Null^, and tamoxifen-treated *Lrig1*^Null^; *Lrig3*^fl/+^ and *Lrig1*^Null^; *Lrig3*^fl/fl^ mice. β-actin was used as a loading control. Immunoblotting was performed 3 days after the last dose of corn oil or TAM. (*C*) Bubble heatmap plot showing scRNA-seq gene expression of markers used to identify the indicated cell state or activity. (*D*) Representative IF images of SI tissue from untreated WT mice, corn oil–treated *Lrig1*^Null^ mice, and TAM-treated *Lrig1*^Null^; *Lrig3*^fl/+^ and *Lrig1*^Null^; *Lrig3*^fl/fl^ mice stained for HES1. The *last columns* of (*A*) and (*D*) are merged images with DAPI. IF of intestinal tissue in (*A*) and (*D*) was done as described in Figure 2. Scale bar, 50 μm.

Injury-induced loss of ISCs is known to trigger a damage response that involves activation of reserve stem cell populations.^43^ We use the term reserve to encompass populations of stem cells dedicated to replenishing highly proliferative Lgr5^+^ cells lost during injury. These include revival stem cells (revSCs) that appear post-injury and are marked by Clusterin (*Clu*) expression^37^^,^^38^ and the recently identified persister intestinal stem cells (pISCs).^40^ Consistent with this, we see increased expression of *Clu* in the ISC population from tamoxifen-treated *Lrig1*^Null^; *Lrig3*^fl/+^ and *Lrig1*^Null^; *Lrig3*^fl/fl^ mice (Figure 13*A* and *B*). Accompanying an increased expression of *Clu*, we observed an increased expression of *Ascl2* (Figure 13*A* and *B*), another marker associated with a damage response and intestinal crypt regeneration.^41^ Unlike revSCs, however, pISCs are present under homeostatic conditions and expand postinjury.^39^ Indeed, increased expression of the pISC gene signature within the larger enterocyte cluster was observed along with expression of *Tob2* and *Emp1*, which mark pISCs^39^ (Figure 13*A* and *B*). These data are consistent with the activation of reserve stem cell populations in response to damage caused by *Lrig3* loss in *Lrig1*^Null^ ISCs.

### LRIG3 Loss in the Absence of LRIG1 Decreases EGFR/WNT Signaling

Multiple studies have shown LRIG1 to negatively regulate EGFR and downstream signaling.^3^^,^^6^^,^^11^ Moreover, studies show that EGFR, along with WNT signaling, is critical to regulating the maintenance and differentiation of crypt ISCs under homeostatic conditions.^3^^,^^11^^,^^44^, ^45^, ^46^ Consistent with this negative role,^3^ the homozygous loss of *Lrig1* resulted in increased EGFR by immunoblotting (Figure 14*B*) and increased EGFR activity as determined by phosphorylated tyrosine (pY)-1068 IF (Figure 14*A*) in *Lrig1*^Null^ small intestinal crypts. Likewise, increased EGFR levels by immunoblotting were also observed in *Lrig1*^Null^ colonic crypts, relative to WT mice (Figure 15*B*). Homozygous deletion of *Lrig3* in *Lrig1*^Null^ intestinal crypts resulted in reduced EGFR protein and activity (pY-1068) in marked contrast to loss of *Lrig1* alone (Figures 14*A* and *B*; 15*A*, *B* and *D*; and 16). *Lrig3* deletion in *Lrig1*^Null^ ISCs significantly reduced ERBB2 protein levels and activity (pY-1221/1222), as well as ERBB3 protein levels (Figures 14*B*, 15*B*, 17*A–D*, and 18*A* and *B*). In line with these results, gene expression analysis showed a decreased expression of both *Erbb2* and *Erbb3* (Figures 14*C* and 15*C*). Although a decreased expression of *Egfr* was not seen, there was an increased expression of several markers associated with post-translational degradation of the ErbB family (*Cbl*, *Grb2*, *Clta*, *Flot2*) (Figures 14*C* and 15*C*). Increased expression of both *Met* and *Ret* was detected following *Lrig3* loss (Figures 14*C* and 15*C*), which has been implicated in bypassing EGFR signaling during EGFR inhibition.^47^ A significant reduction in ERK phosphorylation, downstream of EGFR activation,^48^ was also observed following the heterozygous or homozygous deletion of *Lrig3* in *Lrig1*^Null^ small intestinal crypts (Figure 14*B*).

**Figure 15:**
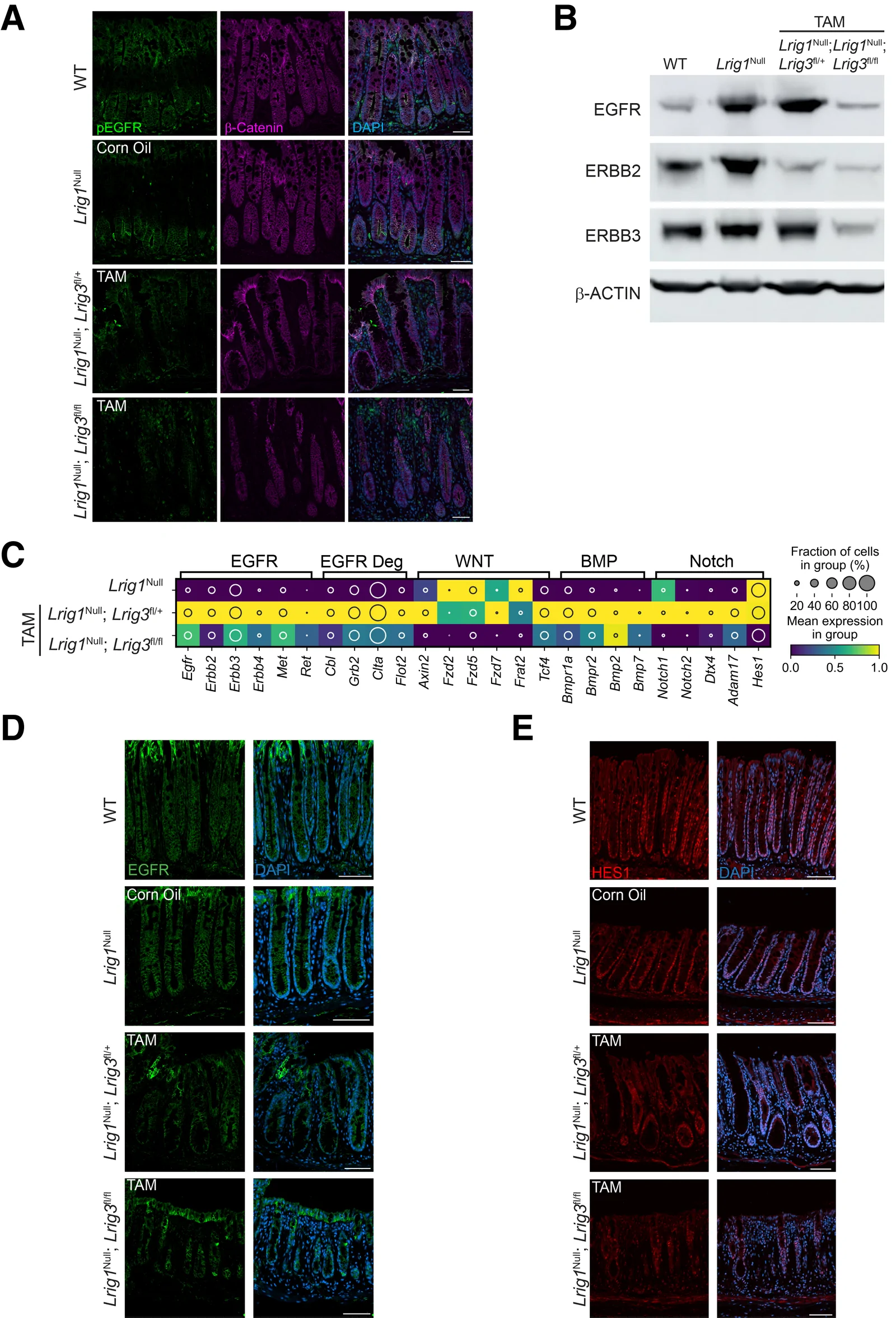
**Tamoxifen-induced deletion of *Lrig3* in *Lrig1*^Null^ cells results in decreased EGFR, WNT, and NOTCH signaling in Colon.** (*A*) Representative IF images of colon tissue from untreated WT mice, corn oil–treated *Lrig1*^Null^ mice, and tamoxifen (TAM)-treated *Lrig1*^Null^; *Lrig3*^fl/+^ and *Lrig1*^Null^; *Lrig3*^fl/fl^ mice stained for β-catenin and phosphorylated tyrosine (pY-1068) EGFR. (*B*) Immunoblotting of the indicated proteins in lysates of isolated crypts from WT mice, corn oil–treated *Lrig1*^Null^ mice, and TAM-treated *Lrig1*^Null^; *Lrig3*^fl/+^ and *Lrig1*^Null^; *Lrig3*^fl/fl^ mice. β-actin was used as a loading control. Immunoblotting was performed 3 days after the last dose of corn oil or TAM. (*C*) Bubble heatmap plot showing the scRNA-seq gene expression of markers used to identify the indicated cell state or activity. Representative IF images of colon tissue from untreated WT mice, corn oil–treated *Lrig1*^Null^ mice, and TAM-treated *Lrig1*^Null^; *Lrig3*^fl/+^ and *Lrig1*^Null^; *Lrig3*^fl/fl^ mice stained for (*D*) EGFR or (*E*) HES1. The *last columns* of (*A*), (*D*) and (*E*) are merged images with DAPI. IF of intestinal tissue in (*A*), (*D*) and (*E*) was done as described in Figure 2. Scale bar, 50 μm.

**Figure 16:**
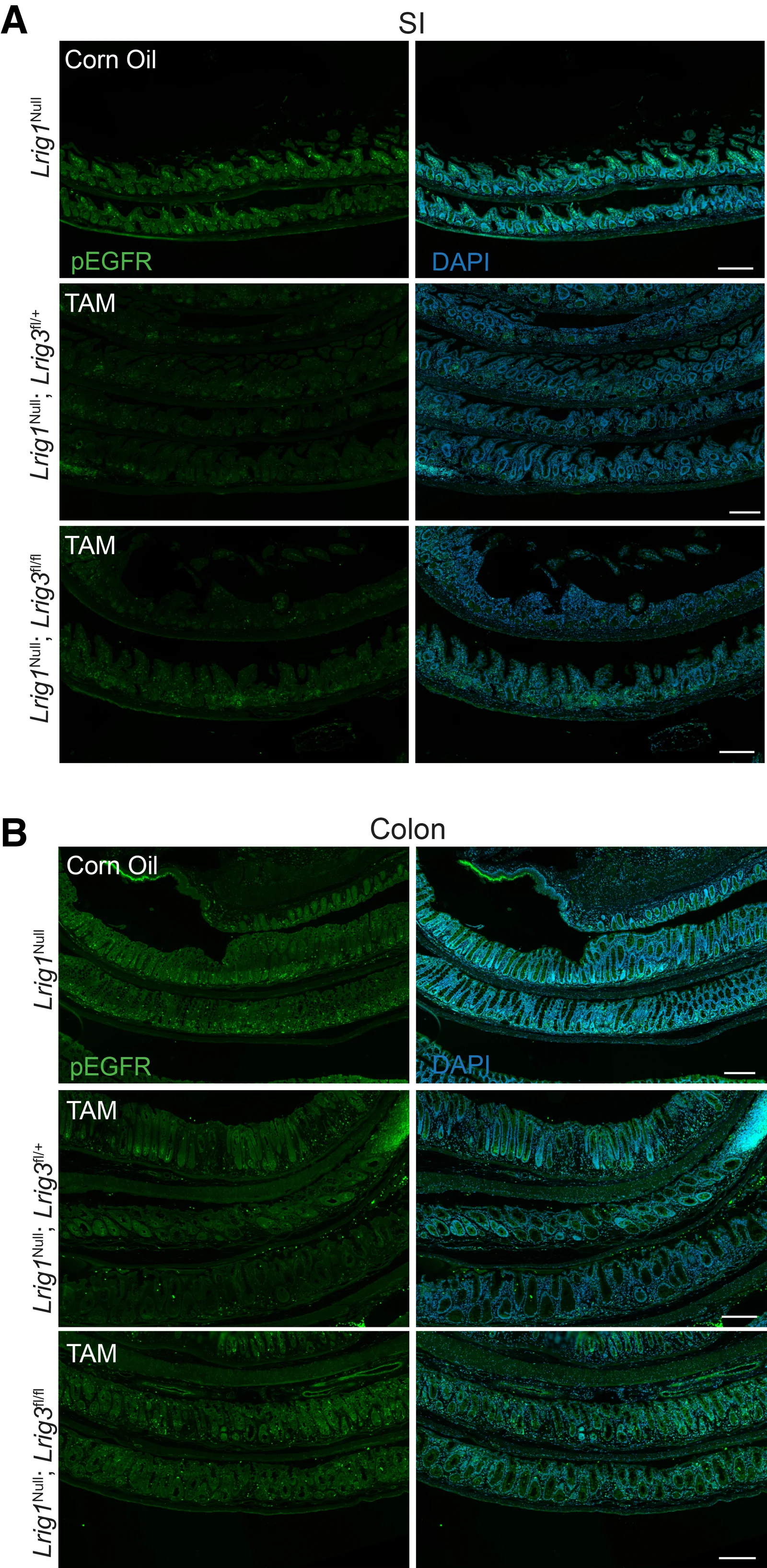
**The loss of LRIG3 in *Lrig1*^Null^ cells decreased EGFR phosphorylation throughout the small intestine (SI) and colon.** Representative IF images showing pY-1068 EGFR signal across a large region of (*A*) SI and (*B*) colon, isolated and analyzed as in Figure 2, from corn oil–treated *Lrig1*^Null^ mice, and tamoxifen (TAM)-treated *Lrig1*^Null^; *Lrig3*^fl/+^ and *Lrig1*^Null^; *Lrig3*^fl/fl^ mice. Scale bar, 200 μm.

**Figure 17:**
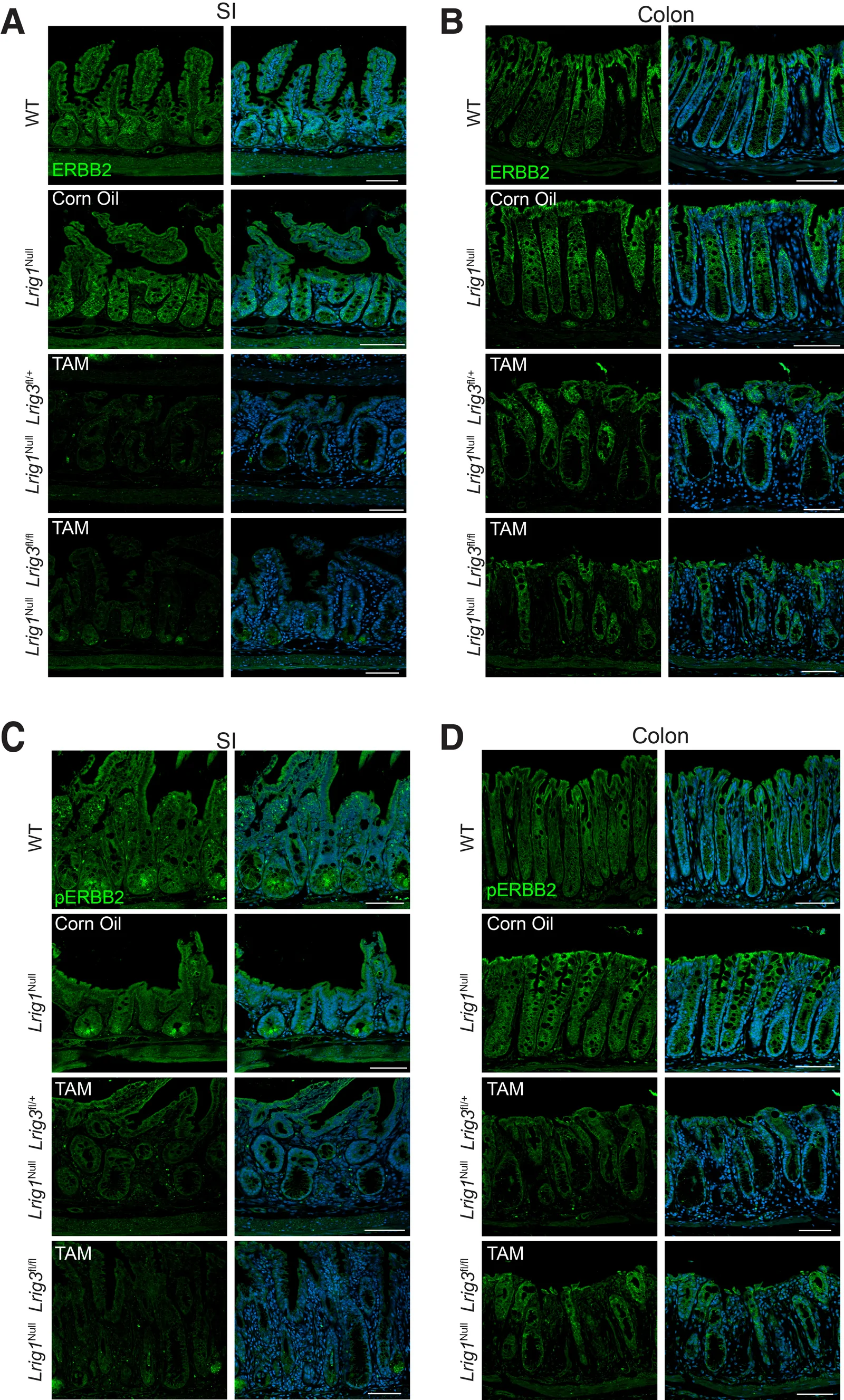
**The decrease in ERBB2 and phospho-ERBB2 in intestinal crypts following the deletion of *Lrig3* in *Lrig1*^Null^ mice.** Representative IF of small intestinal and colonic tissue from corn oil–treated *Lrig1*^Null^ mice and tamoxifen (TAM)-treated *Lrig1*^Null^; *Lrig3*^fl/+^ and *Lrig1*^Null^; *Lrig3*^fl/fl^ mice stained for (*A* and *B*) ERBB2 or (*C* and *D*) pERBB2 (pY-1221/1222). IF analysis was performed as described in Figure 2. Scale bar, 50 μm.

**Figure 18:**
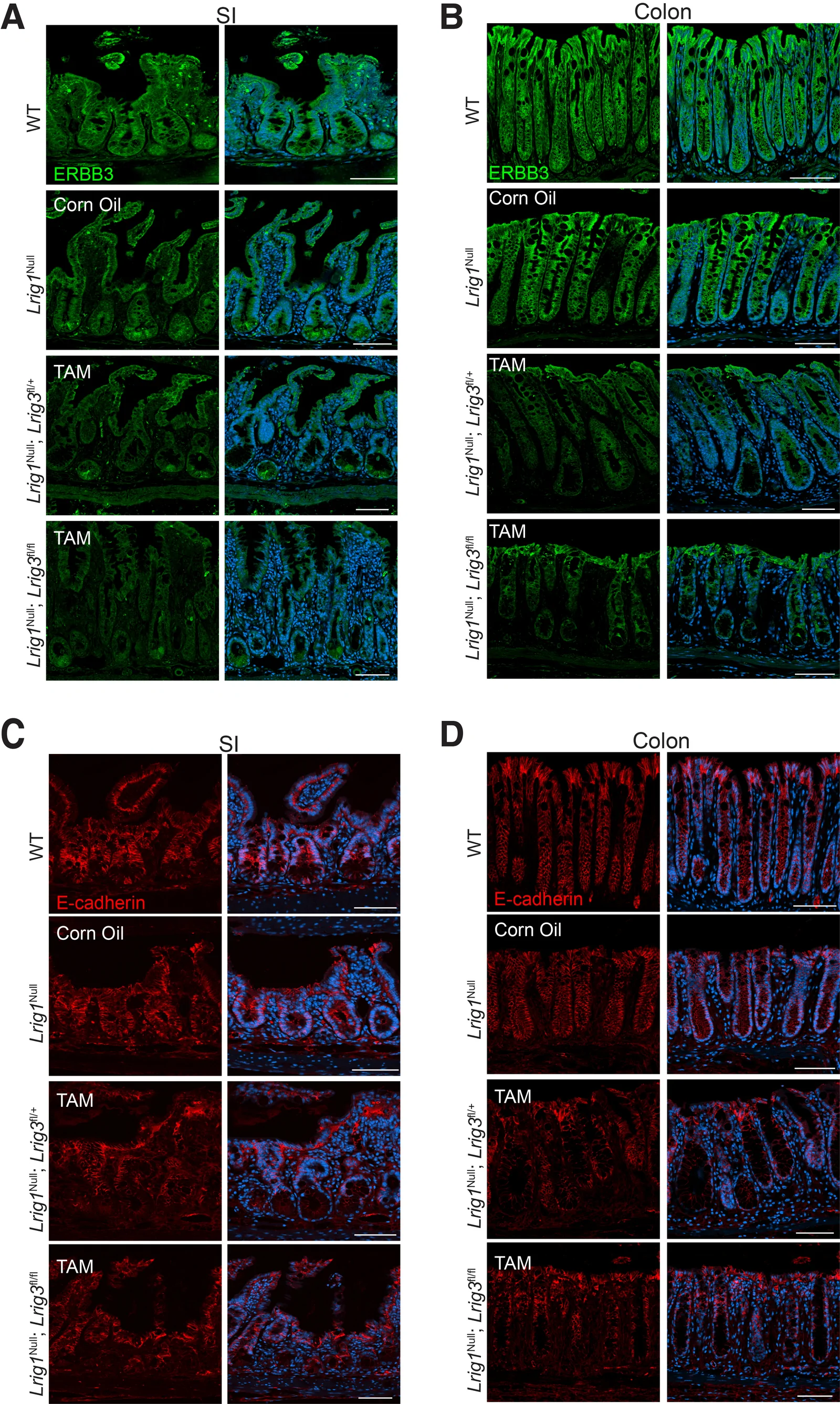
**The decrease in ERBB3 and E-cadherin in intestinal crypts following the deletion of *Lrig3* in *Lrig1*^Null^ mice.** Representative IF of small intestinal (SI) and colonic tissue from untreated WT mice, corn oil–treated *Lrig1*^Null^ mice and tamoxifen (TAM)-treated *Lrig1*^Null^; *Lrig3*^fl/+^ and *Lrig1*^Null^; *Lrig3*^fl/fl^ mice stained for (*A* and *B*) ERBB3 or (*C* and *D*) E-cadherin. IF analysis was performed as described in Figure 2. Scale bar, 50 μm.

*Lrig1* deletion resulted in increased β-catenin levels in small intestinal crypts, as expected, given the established crosstalk between EGFR and WNT signaling (Figure 14*A*). However, heterozygous and homozygous *Lrig3* deletion in *Lrig1*^Null^ intestinal crypts triggered a drastic decrease in β-catenin, indicating decreased WNT signaling (Figures 14*A* and *B* and 15*A*). E-cadherin levels also decreased following the deletion of *Lrig3* in *Lrig1*^Null^ cells in both the small and large intestinal crypts (Figure 18*C* and *D*). Using scRNA-seq data to analyze changes to WNT signaling, we observed an increased expression of *Axin2* and a decreased expression of *Fzd2*, *Fzd5*, and *Frat2*, consistent with decreased Wnt signaling (Figures 14*C* and 15*C*). In addition to EGFR and WNT signaling, BMP and Notch signaling play critical roles in ISC maintenance and secretory cell differentiation.^49^, ^50^, ^51^, ^52^ BMP signaling restricts ISC identity while promoting Paneth and goblet cell maturation.^49^^,^^50^ Conversely, Notch signaling promotes ISC identity and restricts secretory cell differentiation.^51^^,^^52^ We observed that the loss of *Lrig3* in *Lrig1*^Null^ ISCs resulted in an increased expression of BMP markers (*Bmpr1a*, *Bmpr2*, *Bmp2*, *Bmp7*) and Notch ligands**,** often expressed by Paneth cells (*Dll1* and *Dll4*), while showing decreased expression of the Notch signaling marker *Hes1* (Figures 9*A*, 10*A*, 14*C*, and 15*C*), indicating a decrease in Notch signaling in the *Lrig3* heterozygous situation that is exacerbated with the homozygous loss of *Lrig3*. Decreased expression of *Hes1* in the crypts of tamoxifen-treated *Lrig1*^Null^; *Lrig3*^f^^l^^/+^ and *Lrig1*^Null^; *Lrig3*^f^^l^^/f^^l^ mice were also confirmed by IF analysis (Figures 14*D* and 15*E*). These results suggest that the loss of LRIG3 in the *Lrig1*^Null^ ISCs negatively affects EGFR/ERK, leading to decreased WNT signaling and the disruption of other signaling pathways critical to ISC maintenance, differentiation, and crypt homeostasis. These data are consistent with a model in which LRIG3 acts in an opposing manner to balance EGFR levels and downstream signaling. When *Lrig1* is lost, EGFR and Wnt signaling increase; however, the loss of *Lrig3* in this setting counteracts this effect.

### Deletion of *Lrig3* in *Lrig1*^CreER/+^; *Apc*^fl/+^ Mice Markedly Reduces Colonic Tumor Formation

Given the importance of increased EGFR and WNT signaling in the pathogenesis of colonic neoplasia, we hypothesized that *Lrig3* loss in *Lrig1*^CreERT2/+^; *Apc*^fl/+^ mice would reduce EGFR signaling, leading to attenuation of the highly penetrant tumor phenotype observed upon tamoxifen-induced elimination of 1 *Apc* allele in *Lrig1*-expressing cells.^3^^,^^28^ *Lrig1*^CreERT2/+^; *Apc*^fl/+^ and *Lrig1*^CreERT2/+^; *Apc*^fl/+^; *Lrig3*^fl/+^ littermates were injected i.p. with 3 consecutive daily 2-mg doses of tamoxifen (Figure 19*A*). As expected, multiple highly dysplastic distal colonic adenomas were observed in all *Lrig1*^CreERT2/+^; *Apc*^fl/+^ mice approximately 100 days after the last tamoxifen injection (Figure 19*B–D*).^3^^,^^28^ However, the loss of a single allele of *Lrig3* was sufficient to dramatically reduce the tumor formation in 2 separate cohorts (Figures 19*B–D*, 20*A*). As expected, we observed high levels of both EGFR (pY-1068) and β-catenin in both the tumors and the surrounding tissue of the distal colon from tamoxifen-treated *Lrig1*^CreERT2/+^; *Apc*^fl/+^ mice (Figures 19*E* and 20*B*). Moderately high levels of LRIG3 were also observed in the tumor tissue from tamoxifen-treated *Lrig1*^CreERT2/+^; *Apc*^fl/+^ mice (Figure 19*F*). In marked contrast, the entire distal colon region of tamoxifen-treated *Lrig1*^CreERT2/+^; *Lrig3*^fl/+^; *Apc*^fl/+^ mice showed much lower levels of EGFR (pY-1068), β-catenin, and LRIG3 (Figures 19*E* and *F* and 20*C*). Deletion of a single *Lrig3* allele in *Lrig1*^CreERT2/+^; *Apc*^fl/+^ also reduced tumor formation in the small intestine with a trend towards significance. These results demonstrate that the loss of *Lrig3* in *Lrig1*-expressing cells was sufficient to counteract the Lrig1-dependent activation of EGFR, leading to a marked reduction in tumor formation.

**Figure 19:**
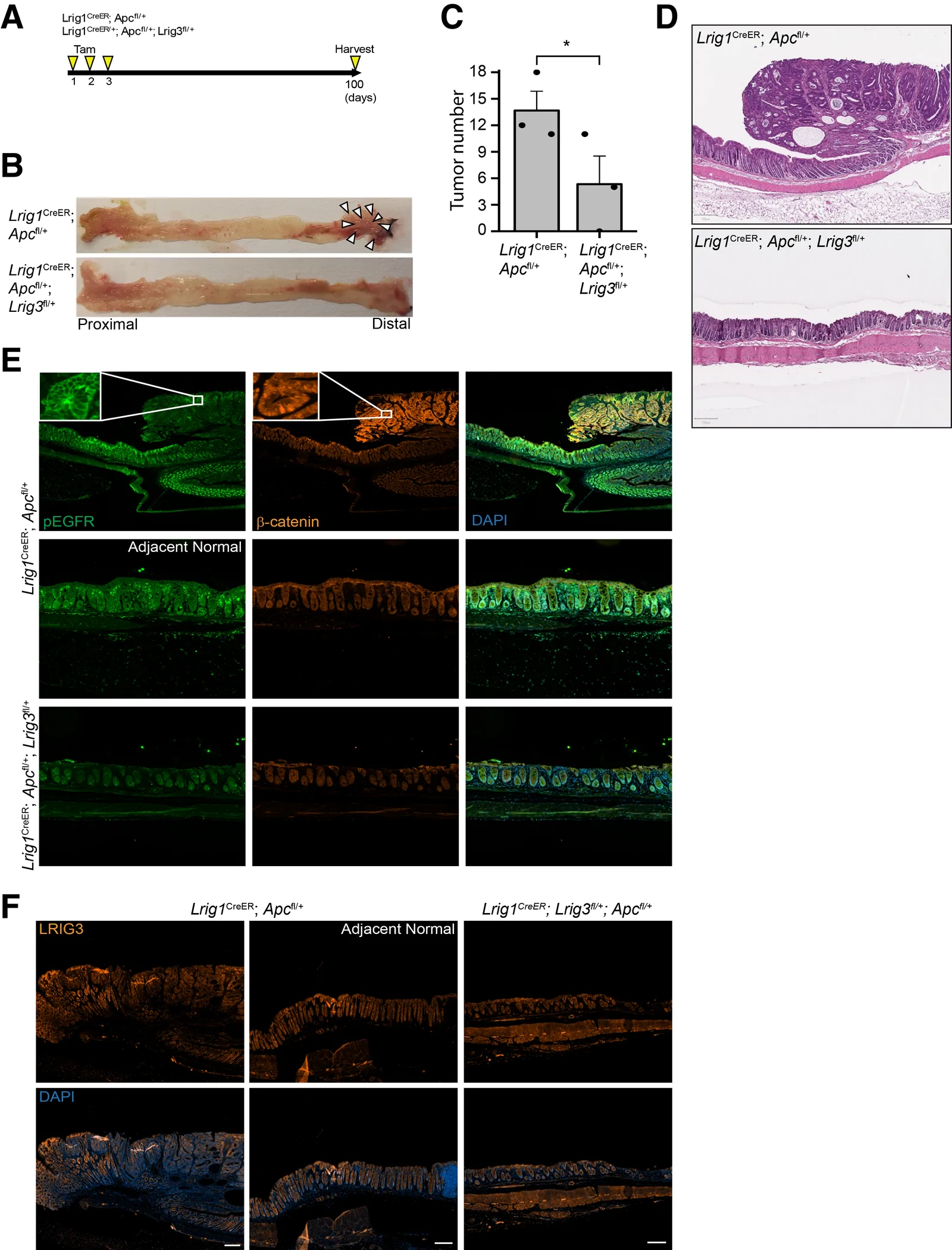
**The deletion of *Lrig3* in tamoxifen (TAM)-treated *Lrig1*^CreER/+^; Apc^fl/+^ mice abrogates tumor formation.** (*A*) Schematic showing the TAM treatment schedule and the harvesting scheme conducted on *Lrig1*^CreER/+^; *Apc*^fl/+^ and *Lrig1*^CreER/+^; *Apc*^fl/+^; *Lrig3*^fl/+^ mice. (*B*) Images of flayed open colons from TAM-treated *Lrig1*^CreER/+^; *Apc*^fl/+^ and *Lrig1*^CreER/+^; *Apc*^fl/+^; *Lrig3*^fl/+^ mice. *White arrowheads* indicate distal colonic tumors. (*C*) Quantification indicating the average number of tumors present in TAM-treated *Lrig1*^CreER/+^; *Apc*^fl/+^ and *Lrig1*^CreER/+^; *Apc*^fl/+^; *Lrig3*^fl/+^ mice (N = 3 mice per genotype). Error bars represent standard error of the mean. ∗*P* < .05 by *t* test. (*D*) H&E staining of distal colonic tissue from TAM-treated *Lrig1*^CreER/+^; *Apc*^fl/+^ (*top*) and *Lrig1*^CreER/+^; *Apc*^fl/+^; *Lrig3*^fl/+^ (*bottom*) mice. Representative images depicting (*E*) pY-1068 EGFR and β-catenin or (*F*) LRIG3 IF in the distal colon of mice treated with TAM.

**Figure 20:**
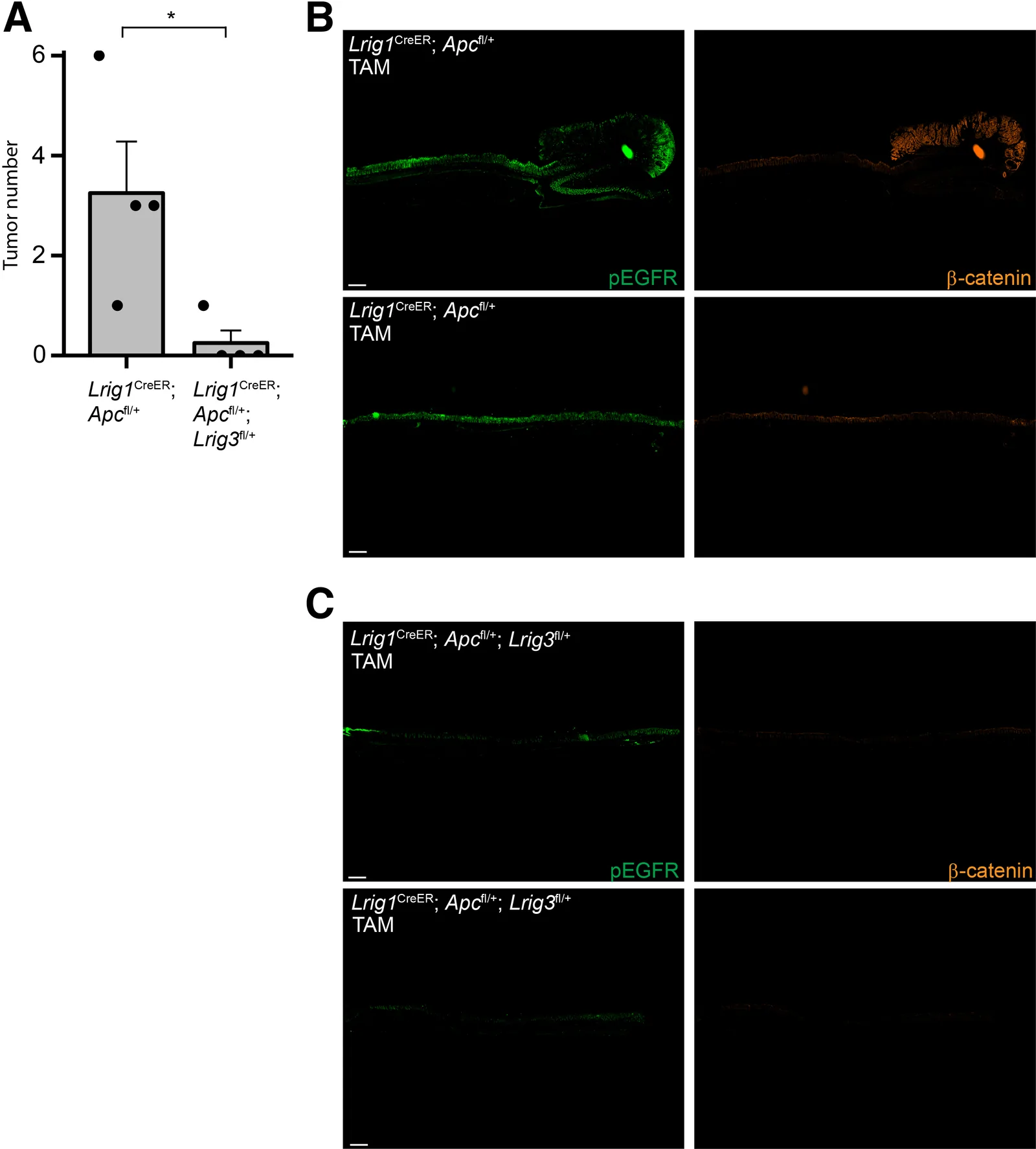
**The deletion of *Lrig3* in tamoxifen-treated *Lrig1*^CreER/+^; *Apc*^fl/+^ mice caused a widespread decrease in EGFR and WNT signaling.** (*A*) Quantification indicating the average number of tumors present in TAM-treated *Lrig1*^CreER/+^; *Apc*^fl/+^ and *Lrig1*^CreER/+^; *Apc*^fl/+^; *Lrig3*^fl/+^ mice (N = 3 mice per genotype). Error bars represent standard error of the mean. ∗*P* < .05 by *t* test. IF images depicting the distal colonic region of (*B*) TAM-treated *Lrig1*^CreER/+^; *Apc*^fl/+^ tissue and (*C*) TAM-treated *Lrig1*^CreER/+^; *Apc*^fl/+^; *Lrig3*^fl/+^ stained with pY-1068 EGFR and β-catenin. In (*B*), the *top row* depicts pY-1068 EGFR and β-catenin levels in a distal colonic tumor; the *bottom row* depicts the same staining in an adjacent normal region. (*C*) Shows regions of the colon analogous to (*B*). Scale bar, 500 μm.

## Discussion

These studies address whether LRIG3 acts to cooperate with or oppose the effects of LRIG1 on EGFR in mouse intestinal crypts. Although LRIG1 is known to be a pan-ERBB negative regulator that is expressed in ISCs at the base of intestinal crypts, we previously reported that EGFR is the major target of LRIG1 in the mouse intestine,^3^^,^^9^^,^^11^^,^^28^ leading us to focus on EGFR and downstream signaling. There are multiple studies showing that LRIG3 can, depending on context, act redundantly or in opposition to LRIG1 as it relates to EGFR regulation.^22^^,^^23^^,^^26^^,^^27^^,^^53^ We generated an *Lrig3* floxed allele and crossed these mice to *Lrig1*^CreERT2/CreERT2^ (*Lrig1*^Null^) mice to inducibly delete *Lrig*3 in *Lrig1*-expressing cells. We reasoned that if LRIG3 cooperates with LRIG1 to negatively regulate EGFR, then its loss would exacerbate the increase in EGFR seen following the loss of *Lrig1*. However, if LRIG3 opposes LRIG1, then its loss would reverse the increase in EGFR triggered by *Lrig1* loss. Herein, we show that heterozygous or homozygous deletion of *Lrig3* in *Lrig1*^Null^ cells reversed the effect of loss of *Lrig1* on EGFR, both in normal physiology and neoplasia.

At homeostasis, Lrig3 antagonizes the effect of Lrig1 on EGFR levels. scRNA-seq analysis showed an increased expression of several markers consistent with the internalization and degradation of EGFR, with our most conclusive evidence being decreased EGFR IF at the crypt base. However, we did not observe a decrease in total EGFR by immunoblotting of isolated tamoxifen-treated *Lrig1*^Null^; *Lrig3*^fl/+^ colonic crypts. This discrepancy likely reflects the restriction of *Lrig1* expression to the crypt base^3^^,^^11^ and the broader distribution of *Lrig3* higher up the crypt axis.^13^ Thus, the deletion of *Lrig3* in *Lrig1*^Null^ ISCs may preferentially affect the crypt base while leaving the upper crypt regions relatively spared. Of additional interest, an increased expression of *ErbB3* has been shown to occur in response to the loss or inhibition of EGFR signaling.^9^^,^^54^ Consistent with this, ERBB3 protein levels were less affected when compared with the near complete loss of EGFR or ERBB2 following *Lrig3* deletion in *Lrig1*^Null^ intestinal crypts. Although we cannot exclude the effects of LRIG3 on other RTKs, our data are consistent with previous work supporting an LRIG1-opposing, EGFR-stabilizing role for LRIG3.

Although the extracellular domains of murine LRIG1 and LRIG3 share 55% identity, the intracellular domains are more divergent. We speculate that the divergent cytoplasmic tails may explain LRIG3-mediated EGFR stabilization, at least in part, by lack of a CBL domain in the cytoplasmic tail of LRIG3.^26^^,^^27^ In LRIG1, the CBL domain has been shown to play a role in recruiting E3 ubiquitin ligases, which, in turn, promotes the degradation of RTKs.^5^^,^^8^^,^^55^ Moreover, although LRIG1 and LRIG3 both have SH3 domains, which facilitate protein binding to the intracellular tail of EGFR,^26^^,^^56^ LRIG3 has an additional SH2 domain that has been shown to facilitate protein binding to tyrosine-phosphorylated EGFR and prevent its dephosphorylation.^57^^,^^58^ Altogether, these data support a model in which the relative balance of the opposing effects of LRIG1 and LRIG3 on EGFR modulate levels of activated EGFR and downstream signaling.

We performed scRNA-seq analysis and immunoblotting to better understand the effects of loss of *Lrig1* and *Lrig3* on EGFR-related signaling pathways. In addition to EGFR signaling, WNT signaling is known to play a critical role in regulating the maintenance and differentiation of ISCs during crypt homeostasis.^45^^,^^46^ *Lrig1* homozygous deletion caused a modest increase in β-catenin levels in small intestinal crypts, hinting at an increase in WNT signaling. The subsequent deletion of Lrig3 in *Lrig1*^Null^ ISCs, however, triggered a significant reduction in β-catenin levels at the bottom of the intestinal crypts isolated from tamoxifen-treated *Lrig1*^Null^; *Lrig3*^fl/+^ and *Lrig1*^Null^; *Lrig3*^fl/fl^ mice. The loss of β-catenin was accompanied by a significant decrease in ERK phosphorylation, indicating a loss of activity in the EGFR-RAS-ERK pathway. This is consistent with data showing cross regulation between WNT and EGFR-RAS-ERK signaling.^59^, ^60^, ^61^, ^62^ A marked decrease in E-cadherin levels was also observed upon the inducible deletion of *Lrig3* in *Lrig1*^Null^ intestinal cells, which is not unexpected, given the role that E-cadherin/β-catenin membrane complexes have in modulating WNT signaling and regulating epithelial cell integrity.^63^ Here, we show that *Lrig3* deletion in *Lrig1*^Null^ ISCs drastically affects WNT signaling along with EGFR-RAS-ERK signaling, leading to decreased proliferation, increased cell death, and increased crypt loss. Tight control over WNT and EGFR-RAS-ERK signaling is necessary to ensure that intestinal crypt homeostasis is well-regulated. Collectively, these data establish LRIG1 and LRIG3 as critical regulators of intestinal crypt homeostasis by modulating EGFR and WNT signaling.

In addition to markedly reduced EGFR and WNT signaling**,** we show that the loss of *Lrig3* (either heterozygous or homozygous) in *Lrig1*^Null^ ISCs decreased the overall number of ISCs, their proliferative status, and the expression of multiple ISC markers. Concomitant with the loss of the ISC population, a striking morphologic alteration was widening of the crypt base in both the small intestine and colon with the loss of *Lrig3* in *Lrig1*^Null^ ISCs. In the small intestine, the widened crypt base largely consisted of cells coexpressing both Paneth (*Lyz*, *Mmp7*) and goblet (*Muc2* and *Tff3*) markers. TEM analysis showed the presence of mucin granules with an electron dense core, a defining feature of Paneth/goblet intermediate cells.^35^^,^^64^ Although rare, intermediate cells are thought to be a cell state that occurs prior to Paneth and goblet cell specification; intermediate cells can also appear during cell-fate switching between Paneth and goblet cells.^33^, ^34^, ^35^^,^^64^^,^^65^ Recent work showed that the loss of Gremlin1, a BMP antagonist, drastically alters the balance between WNT and BMP signaling, causing a loss in ISC identity, which is accompanied by the appearance of Paneth/goblet intermediate cells.^33^ It was further shown that WNT^high^ BMP^low^ conditions trigger goblet cells to display Paneth cell characteristics, whereas WNT^low^ BMP^high^ conditions trigger the opposite effect.^33^ These findings are congruent with other studies showing that the inhibition of mitogen-activated protein kinase (MAPK) signaling promotes Paneth cell expansion at the expense of goblet cells, whereas its activation has the opposite effect.^34^ Here, we show that, along with decreased EGFR-RAS-MAPK and WNT signaling, *Lrig3* loss in *Lrig1*^Null^ ISCs resulted in an increased expression of BMP signaling markers, creating a WNT^low^, BMP^high^, EGFR-RAS-MAPK^low^ environment. This environment, considering previous studies, would lead to an increase in Paneth/goblet intermediate cells at the expense of ISCs. Our finding that *Lrig1*^Null^ crypts with heterozygous *Lrig3* loss lack WT Paneth cells is also consistent with studies showing that this signaling environment leads to post-mitotic secretory cell fate switching.^33^ Although goblet cells are present, colonic crypts lack lysozyme^+^ Paneth cells and thus do not have classically defined Paneth/goblet intermediate cells. Recent studies, however, suggest that REG4^+^ DSCs are the Paneth cell equivalent in the colon.^36^ In further support of this, loss of *Lrig3* in *Lrig1*^Null^ colonic ISCs caused a marked increase in REG4^+^ DSCs that, like Paneth/goblet intermediate cells, coexpress goblet cell markers. Consistent with an overall reduction in stem cell identity and increased aberrant differentiation, both the small intestinal and colonic crypts showed increased expression of several cell-cycle exit and senescence markers, including β-galactosidase, following the loss of *Lrig3* in *Lrig1*^Null^ ISCs. Additionally, the homozygous loss of *Lrig3* in *Lrig1*^Null^ ISCs increased the number of small intestinal and colonic crypt cells positive for γ-H2AX, a marker indicative of unrepaired DNA damage due to long-term cellular senescence.^66^ Altogether, these results reinforce our model that LRIG1 and LRIG3 interact antagonistically to preserve ISC self-renewal and influence differentiation by regulating EGFR-RAS-ERK and WNT signaling.

Multiple studies show activation of reserve stem cell populations as key to intestinal crypt regeneration following injury and the loss of the ISC population.^37^^,^^43^^,^^67^ As noted in the Results, reserve stem cell populations encompass both revSCs and pISCs. The damage-induced activation of quiescent revSCs, whose dedifferentiation is critical to crypt regeneration, is marked by the increased expression of *Clu*.^37^ Additional work shows that the transcription factor ASCL2 and its downstream target genes are critical to this dedifferentiation response.^41^ The increased expression of *Clu* and *Ascl2* in the intestinal crypts following *Lrig3* loss in *Lrig1*^Null^ cells suggests the activation of revSCs in response to damage linked to reduced EGFR and WNT signaling and depletion of ISCs. Recent work from our lab has characterized pISCs, a novel population of stem cells marked by *Tob2* expression that are specified early in embryogenesis, persist into adulthood, and are critical to crypt regeneration following injury.^39^ In that prior work, pISCs were identified in other datasets via a gene signature derived from characterization of this population.^39^ In line with this, we show that *Lrig3* loss in *Lrig1*^Null^ ISCs caused expansion of pISCs to include a larger portion of enterocytes, with their further expansion into the stem cell cluster as well as the Paneth and goblet cell clusters. We also see increased *Emp1* (a gene in the pISC gene signature) expression that follows the pISC population expansion pattern, from enterocytes through the stem cell cluster and into the Paneth and goblet cell cluster. Previously, we showed that loss of TOB2 or *Tob2*-expressing pISCs significantly disrupted damage-induced crypt regeneration.^39^ Moreover, ASCL2, a binding partner of TOB2, has previously been shown to promote activation of the dedifferentiation pathways necessary to promote crypt regeneration.^39^^,^^41^ Consistent with this, *Tob2* and *Ascl2* expression increased in stem cell populations from tamoxifen-treated small intestinal and colonic samples. These data indicate that the damage following the deletion of *Lrig3* in *Lrig1*^Null^ ISCs triggers intestinal crypt regeneration via the activation of reserve stem cell populations. The impaired ISC maintenance and aberrant differentiation with heterozygous *Lrig3* loss and the crypt loss with homozygous *Lrig3* loss, strongly suggest that, without LRIG1 and LRIG3 to maintain balanced EGFR and WNT signaling, the crypt stem cell niche collapses, and the overall regenerative process is compromised. Our work describes the impact of LRIG1 and LRIG3 on the ISC niche, providing the framework for future mechanistic work at the biochemical and molecular level.

Previous work established *Lrig1* as a tumor suppressor and that its homozygous deletion leads to spontaneous tumor formation in the small intestine, which, in large part, is due to increased EGFR signaling.^3^^,^^9^ The inducible deletion of a single allele of *Apc* in haploinsufficient *Lrig1*^CreER^ mice prompts tumor formation in the distal colon, following the stochastic loss of the second *Apc* allele.^28^ Here, we show that the deletion of a single allele of *Lrig3* in *Lrig1*^CreER/+^; *Apc*^fl/+^ mice significantly attenuates tumor formation along with an overall decrease in EGFR and WNT signaling throughout the distal colon. These findings highlight a critical role of EGFR signaling and perhaps of WNT signaling, in the formation of distal colon tumors. This work suggests that the antagonistic relationship between LRIG1 and LRIG3 on regulating EGFR and downstream signaling maintains ISC homeostasis and impacts colorectal tumor formation and progression.

## Methods and Materials

### Mice and Tamoxifen Injections

All mice were of a mixed background of C57BL/6 and 129S1 and housed in a specific pathogen-free facility with light cycle–controlled conditions. Mice were fed standard rodent lab chow and had ad libitum access to water. All mouse protocols were approved and performed in accordance with the Vanderbilt University Medical Center (VUMC) Animal Care and Use Program. The generation and characterization of the *Lrig1*^CreERT2^ mouse line was previously described in.^3^ To generate the *Lrig3*^fl^ allele, the *129 Lrig3* BAC clone was obtained from the Sanger institute, and BAC recombineering was used to construct a targeting vector that placed LoxP sites 5′ and 3′ of exons 4 to 12 and a PGK-Neo cassette (Figure 1*C*). Homologous recombination was used to modify the native *Lrig3* locus in embryonic stem cells, which were injected into blastocyst stage *129* mouse embryos by the Transgenic Mouse/ES Cell Shared Resource at Vanderbilt University. The resulting chimeric mice were bred to *FlpE* mice to remove the PGK-Neo cassette. To induce recombination of the *Lrig3*^fl^ allele, *Lrig1*^CreERT2/CreERT2^ (*Lrig1*^null^), *Lrig1*^null^; *Lrig3*^fl/+^, and *Lrig1*^null^; *Lrig3*^fl/fl^ mice (age, 8–30 weeks) underwent three daily consecutive i.p. injections of either corn oil or 2 mg of tamoxifen (Sigma). Unless otherwise noted, *Lrig1*^null^ and *Lrig1*^null^; *Lrig3*^fl/+^ mice were dissected 7 days post final tamoxifen injection. Tamoxifen-injected *Lrig1*^null^; *Lrig3*^fl/fl^ mice became noticeably lethargic and emaciated 4 to 5 days after the final tamoxifen injections, necessitating their dissection at 4 days post final tamoxifen injections. To induce tumor formation, Lrig1CreER/+; Apcfl/+ and Lrig1CreER/+; Apcfl/+; Lrig3fl/+ mice (age 9-30 weeks) were given 3 daily consecutive doses of 2 mg of tamoxifen. The mice were euthanized approximately 100 days after the final tamoxifen injection. Tumor growth was monitored by colonoscopy using the Karl Storz Endoscope system under appropriate anesthesia with isoflurane (Piramal Critical Care Inc). Roughly equal numbers of male and female mice were used for this study.

### EdU Injections and Detection

For EdU treatments, corn oil–treated *Lrig1*^null^ and tamoxifen-treated *Lrig1*^null^; *Lrig3*^fl/+^, and *Lrig1*^null^; *Lrig3*^fl/fl^ mice were given a single i.p. injection of EdU (50 mg/kg; Invitrogen) 4 hours prior to dissection. Dissections were performed 7 days (*Lrig1*^Null^ and *Lrig1*^Null^; *Lrig3*^fl/+^ mice) and 4 days (*Lrig1*^Null^; *Lrig3*^fl/fl^ mice) after the last dose of tamoxifen. Detection of EdU was performed using the Click-IT Plus EdU Alexa Fluor 555 Imaging Kit (Invitrogen) according to the manufacturer’s instructions.

### Tissue Preparation and Staining

Following euthanasia, intestinal tissue was removed, flushed with PBS (Corning) and flayed open lengthwise on Whatman filter paper and fixed in 4% paraformaldehyde (ThermoScientific) overnight at 4 °C. Fixed intestinal tissue was washed 3 × 5 minutes in PBS and Swiss rolled. Swiss-rolled tissue was bisected, and one-half was placed in a tissue cassette in 70% ethanol for paraffin embedding, and the other one-half was incubated overnight at 4 °C in 30% sucrose. After sucrose incubation, tissue was embedded in OCT (Fisher). Formalin-fixed paraffin-embedded (FFPE) tissue blocks were generated by the VUMC Translational Pathology Shared Resource (TPSR). The TPSR performed all tissue sectioning and hematoxylin and eosin (H&E) staining. TUNEL staining was performed by the TPSR using immunohistochemistry. H&E and TUNEL immunohistochemistry slides were scanned by the Digital Histology Shared Resource at VUMC (digislide.app.vumc.org), with depicted images generated from the resulting scans. Dehydrated FFPE tissue sections of 7-μm thickness were first rehydrated as follows: 3 × 5-minute incubations in Histoclear (CAT), 3 × 5-minute incubations in 100% ethanol, 2 × 5-minute incubations in 95% ethanol, 1 × 5-minute incubation in 70% ethanol, 1 × 5-minute incubation in 50% ethanol, and then 2 × 5-minute incubations in PBS. The tissue sections were permeabilized for 10 minutes at room temperature in PBS with 0.3% Triton X-100. After washing with PBS (3 × 5 minutes each), the sections underwent antigen retrieval using 10 mM citrate buffer pH 6.0 for 20 minutes at 105 °C in a pressure cooker, followed by a 20-minute incubation at room temperature. Tissue sections were then washed in PBS (3 × 5 minutes each) before blocking for 1 hour at room temperature in PBS with 5% normal donkey serum and 3% bovine serum albumin (BSA). After blocking, the slides were incubated with primary antibodies in PBS with 3% BSA (RPI) (Supplementary Table 1) overnight in a humidifier chamber at 4 °C. The next day, the slides were washed in PBS (3 × 5 minutes each) and incubated with secondary antibodies in 3% BSA with 4′,6-diamidino-2-phenylindole (DAPI) for 1–2 hours at room temperature, before being washed in PBS and mounted. Antibodies with directly conjugated fluorophores were incubated in PBS with 3% BSA overnight after blocking and then washed and mounted the next day. Fluorescence images from Figures 7, 8*A*, 16, 19*E* and *F*, and 20*B* and *C* were generated using an Invitrogen EVOS S1000 Spatial Imaging System; all other fluorescent images were generated using a Nikon A1R laser confocal microscope.

### Colonic Crypt Protein Isolation

Flayed open small intestine or colon tissue (dissected and processed as described above) was cut into ∼1 cm pieces and washed twice in PBS. The pieces were then incubated in an ice-cold solution of 3 mM EDTA (Corning) and ethylene glycol tetraacetic acid (Sigma) with 500 μM dithiothreitol in PBS for 30–45 minutes at 4 °C on a rotator. After incubation, the tissue was allowed to settle down, the supernatant was removed, and ∼10 mL of ice-cold PBS was added. The tissue then underwent vigorous shaking to release the crypts. After shaking, the suspension was collected, and the process of adding 10 mL of PBS with subsequent shaking was repeated 2 more times. The final collection of detached crypts (∼30 mL) was centrifuged at 500 × g for 5 minutes. The supernatant was then removed, and the resulting pellet was resuspended in CelLytic M mammalian cell lysis buffer (Sigma) with protease inhibitor (complete, Mini Protease Inhibitor Cocktail, Roche) and phosphatase inhibitor (PhosSTOP EASYpack, Roche) at a ratio of 1:2. The resuspended crypts were then incubated on ice for 30 minutes before the mix was centrifuged. The resulting supernatant was removed, and the protein concentration was determined using a Pierce BCA kit (ThermoFisher).

### Immunoblotting

Approximately 40–50 μg of protein was diluted in Laemmli buffer (Bio-Rad) with 5% β-mercaptoethanol (Sigma) and boiled for 5 minutes. The boiled samples were then loaded onto a sodium dodecyl sulfate-polyacrylamide gel electrophoresis gel (NuPAGE 4%–12% Bis-Tris precast gel, ThermoFisher) and run at 150V for ∼1.5 hours. The protein was then transferred from the gel to a nitrocellulose membrane at 25V for 2 to 3 hours at room temperature. The membranes were then blocked in 5% milk in Tris-buffered saline (TBS) with 0.1% Tween-20 for 1 hour at room temperature. After blocking, the membranes were incubated with the primary antibody overnight at 4 **°**C in 5% BSA in TBS. The next day, the membranes were washed 3 × 5 minutes each in TBS before incubation with horseradish peroxidase–conjugated secondary antibodies at room temperature for 1 hour. After incubation with the secondary antibody, the membranes were washed, and an enhanced chemiluminescence reagent was added, with the subsequent images captured on an Amersham Imager 680 (GE Healthcare).

### Electron Microscopy

Small 1-mm pieces of intestine were fixed in 2% glutaraldehyde and 2% paraformaldehyde in 0.1 M cacodylate. Following primary fixation, the samples were sequentially postfixed in 1% tannic acid, 1% OsO_4_, and enblock stained in 1% uranyl acetate each for 1 hour, followed by dehydration in a graded ethanol series. The samples were infiltrated with Epon-812 using propylene oxide as a transition solvent, and the resin was polymerized at 60 °C for 48 hours. Ultrathin sections were cut on a Leica UC ultramicrotome at a nominal thickness of 70 nm and stained with 2% uranyl acetate and lead citrate.

The samples were imaged on a JEOL 2100+ TEM operating at 200 keV using an AMT nanosprint 15MKII CMOS camera. Acquisitions were performed using SerialEM, and tilesets were stitched using the etomo/IMOD software suite. Electron microscopy was performed in part through the use of the Vanderbilt Cell Imaging Shared Resource.

### Hybridization Chain Reaction–Fluorescence In Situ Hybridization

OCT-embedded intestinal tissue was sectioned onto glass slides (10 μm). Custom-designed HCR-FISH probes were purchased from Molecular Instruments, Inc, and the HCR Gold RNA-FISH protocol was used to visualize markers using the manufacturer protocols (https://www.molecularinstruments.com/). The resulting slides were incubated in a DAPI solution at room temperature before mounting in Prolong Gold (Invitrogen). Images were generated using Nikon A1R.

### Cell Isolation for Single-Cell RNA Sequencing

Isolated crypts were pelleted and resuspended in 4 ml Dulbecco’s phosphate-buffered saline (DPBS) containing 20 mM HEPES, 1.25 mg/ml DNASE (Sigma DN25), and 2.5 mg/mL cold-active protease (Sigma P5380) for dissociation. Crypts were incubated at 4 °C with gentle inversion for 25 minutes, then triturated 10 to 20 times with a P1000 pipette to mechanically separate cells. Cell suspensions were diluted to 10 mL with DPBS containing 0.02% BSA and spun at ∼500 × g for 5 minutes at 4 °C to pellet, then resuspended in 1 mL of fresh DPBS and 0.02% BSA and passed through a 70-μm filter (Pluriselect) into a new tube. The filtered cell suspensions were washed 2 additional times before being loaded for single-cell encapsulation using PIPseq T2 kits (Fluent Biosciences/Illumina). In summary, ∼5000 cells are loaded into a tube of barcoded beads, then an oil is added along with controlled vortexing to enable emulsification into droplets for capture. Cells are lysed through the addition of a lysis emulsion, and RNA is released and hybridizes to the beads, enabling the barcoding of complementary DNA through reverse transcription. The ore-libraries were amplified and submitted to Vanderbilt Technologies for Advanced Genomics for library construction and sequencing. The libraries were dual-indexed and sequenced on a NovaXP instrument (Illumina), targeting 100M reads each. scRNA-seq was performed on crypts isolated from corn oil–treated *Lrig1*^null^ mice (N = 2) and tamoxifen-treated *Lrig1*^null^; *Lrig3*^fl/+^, and *Lrig1*^null^; *Lrig3*^fl/fl^ mice (N = 2), as well as WT mice (N = 2) for normal colon tissue.

#### Single-cell RNA sequencing data analysis

##### Sequence alignment, raw data filtering, and cell type annotation

FASTQ files were aligned to the mouse reference genome GRCm39 (GENCODE vM29, Ensembl 106) using Fluent BioSciences PIPseeker (v02.01.03) to generate a cellxgene-formatted count matrix. scRNA-seq data quality was assessed using AmbiQuant^68^ with the raw matrices. The matrix was then filtered following the STAR Protocol,^69^ and cells with a Dropkick score >0.5 were retained for downstream analyses. The cell types were annotated based on canonical marker genes of the colon and small intestine.

##### Data processing

For datasets generated from *Lrig1*^null^ mice, cells were randomly subsampled using the scanpy.pp.subsample() function before downstream analyses to ensure the same number of cells across treatment conditions.^70^ Batched effect correction was performed on the *Lrig1*^null^ mice datasets using scvi-tools.^71^ Batch-corrected UMAP representations were generated with the scVI corrected count matrices with scanpy.^70^

##### Gene expression comparison

To compare individual gene and gene signature expression across treatment conditions, cells from each condition were extracted and visualized on the scVI batch-corrected UMAP coordinates. For individual markers, raw count values were visualized on the UMAP as color overlays. For gene signatures, signature scores were computed using the scanpy.tl.score_genes() function^70^ from the arcsinh-transformed counts of gene lists of interest. The scores were visualized on the UMAP as color overlays. pISC genes were queried from.^40^ Intestinal cell type-specific markers were queried from.^30^ Cell-cycle genes were queried from.^72^ Gene expression dot plots were generated using the scanpy.pl.dotplot() function.^70^^,^^73^

To compare cell population changes, cells of each type across treatment conditions were counted and visualized as proportions of the entire cell population of each condition using the seaborn.histplot() function.^73^

##### Quantification and statistics

To obtain the percentage of TUNEL+ cells, at least a 500 mm^2^ area of a TUNEL-stained intestinal Swiss roll section for each condition (N = 3 mice per condition) was assessed for number of TUNEL+ cells present out of the total number of cells in mucosal area (minus the muscularis mucosae and villi) and recorded as a percentage. Total quantification area was comprised of several areas throughout the Swiss roll FFPE section. To quantify the changes in crypt numbers (Figure 4*C*), an H&E section of intestinal Swiss roll for each condition (N = 3 mice per condition) was used to visually count the total number of crypts present in a given length (mm) of intestinal epithelium. Several regions throughout the intestinal epithelium were assessed for a total of ∼6 to 9 mm of intestinal tissue. To quantify the crypt base width, an H&E section of an intestinal Swiss roll was used to quantify the width (in μm) across the thickest part of the bottom of the crypt. The bottom of the crypt was defined as the area of the crypt below the +4 to 5 cell position. A minimum of 50 crypts interspersed throughout the Swiss roll were assessed for each condition (N = 3 mice per condition). All quantifications were done using QuPath. *P* values were calculated using R, with the specific statistical test used indicated in the figure legend.

## Declaration of Generative AI and AI-Assisted Technologies in the Writing Process

During the preparation of this work, the authors used Microsoft’s Copilot feature in order to improve language, clarity, and readability of the text written by the authors. After using this tool, the authors reviewed and edited the content as needed and take full responsibility for the content of the published article.

## Supplementary Material

Supplementary Figure

Extended PDF
