# Supplementary material for: Opposing Effects of LRIG1 and LRIG3 on EGFR Maintain Intestinal Homeostasis and Mitigate Likelihood of Colonic Neoplasia
Source: Cell Mol Gastroenterol Hepatol. 2026 Aug 31;20(12):101873. doi: 10.1016/j.jcmgh.2026.101873 (PMC13677089; doi:10.1016/j.jcmgh.2026.101873)
Supplement: Supplementary file 1 [file mmc1.pdf]

Supplemental Table 1

Antibody for IF	Source	Catalog#
LRIG3	Novus Biologicals	NBP1-81877
LYSOZYME	Dako	A0099
MUC2	Cloud Clon	PAA705mu01
TFF3 eFluor 660	Invitrogen	50-4758-82
KI-67 Alex Fluor 647	BioLegend	652408
SOX9 Alex Fluor 555	Abcam	AB202516
OLFM4	Cell Signaling	39141
β-CATENIN Alexa Fluor 555	Vanderbilt Antibody Core	
Phospho-EGFR Alexa Fluor 488	Abcam	AB205827
EGFR	Abcam	AB52894
REG4	R&D	AF1379
ERBB2	Cell Signaling	2165
Phospho-ERBB2	Cell Signaling	2243
ERBB3	Cell Signaling	12708
E-CADHERIN	BD Transduction	610181
**Antibody for WB**		
β-CATENIN	BD Transduction	610154
EGFR	Millipore	06847
β-Actin	Sigma-Aldrich	A5316
ERBB2	Cell Signaling	4290
ERBB3	Cell Signaling	12708
Phospho-ERK1/2	Cell Signaling	9101
OLFM4	Cell Signaling	39141
**Reagents**		
TAM Sigma Cat# T5648	Sigma-Aldrich	T5648
EdU	Invitrogen	A100044
Sodium Citrate	EMD	SX0445-3
Sucrose	RPI	S24060-1000.0
azoxymethane	Sigma-Aldrich	A5486
Probe: Lrig3	Molecular Instruments	
Amplifier B1-546	Molecular Instruments	NA
Amplifier B3-488	Molecular Instruments	NA
HCR Probe Hybridization	Molecular Instruments	PBH03524
HCR Probe Wash Buffer	Molecular Instruments	BPW03224
HCR Amplification Buffer	Molecular Instruments	BAM03924
4% paraformaldehyde	Thermo Fisher Scientific	J19943-K2
Histoclear	National Diagnostics	HS-200
Triton X-100	Millipore	TX1568-1
Bovine Serum Albumin	RPI	A30075-100.0
Normal Donkey Serum	Jackson Immune Research	017-000121
Prolong Gold AntiFade Reagent	Thermo Fisher Scientific	P36930
Glycerol	RPI	G22020-1.0
0.5M EDTA pH 8	Corning	46-034-CI
EGTA	Sigma-Aldrich	E4378
1M HEPES	Corning	25-060-CI
Dnase I	Sigma-Aldrich	DN25
Protease	Sigma-Aldrich	P5380
10X PBS	Corning	46-013-CM
Dithiothreitol (DTT)	RPI	D11005
CelLytic™ M mammalian cell lysis buffer	Sigma-Aldrich	C2978
complete™, Mini Protease Inhibitor Cocktail	Roche	04693124001

PhosSTOP EASYpack	Roche	04906837001
Pierce BCA kit	Thermo Fisher Scientific	23227
4x Laemmli Sample Buffer	Bio-Rad	1610747
PageRuler™ Plus Prestained Protein Ladder	Thermo Fisher Scientific	26619
Bolt™ Bis-Tris Plus Mini Protein Gels, 4-12%	Invitrogen	NW04120BOX
β-mercaptoethanol	Sigma-Aldrich	M3148
10X TBS	Corning	46-012-CM
PIPseq T2 kits	Fluent Biosciences	
