# Supplementary material for: Opposing Effects of LRIG1 and LRIG3 on EGFR Maintain Intestinal Homeostasis and Mitigate Likelihood of Colonic Neoplasia
Source: Cell Mol Gastroenterol Hepatol. 2026 Aug 31;20(12):101873. doi: 10.1016/j.jcmgh.2026.101873 (PMC13677089; doi:10.1016/j.jcmgh.2026.101873)

Figure 14B Westerns

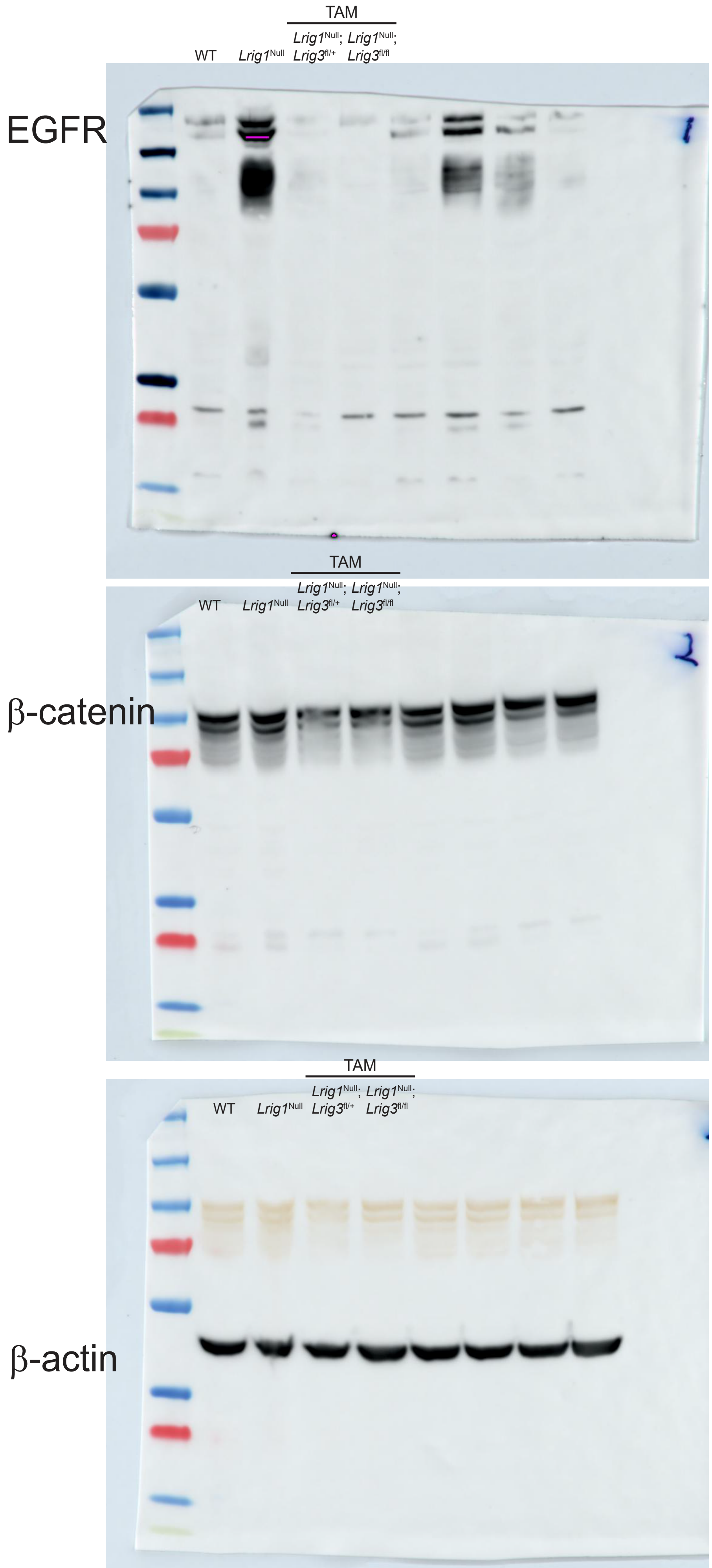

Figure 14B Westerns

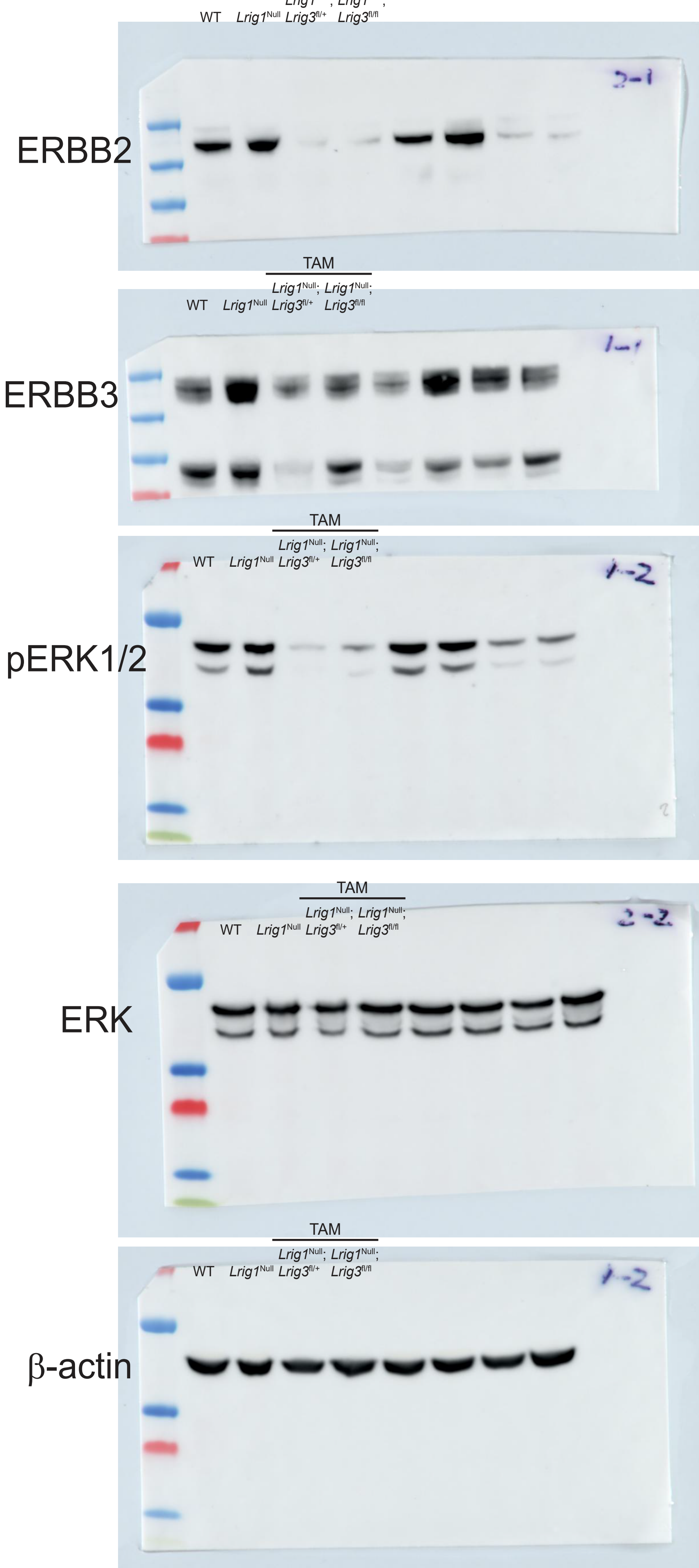

Figure 14B Westerns

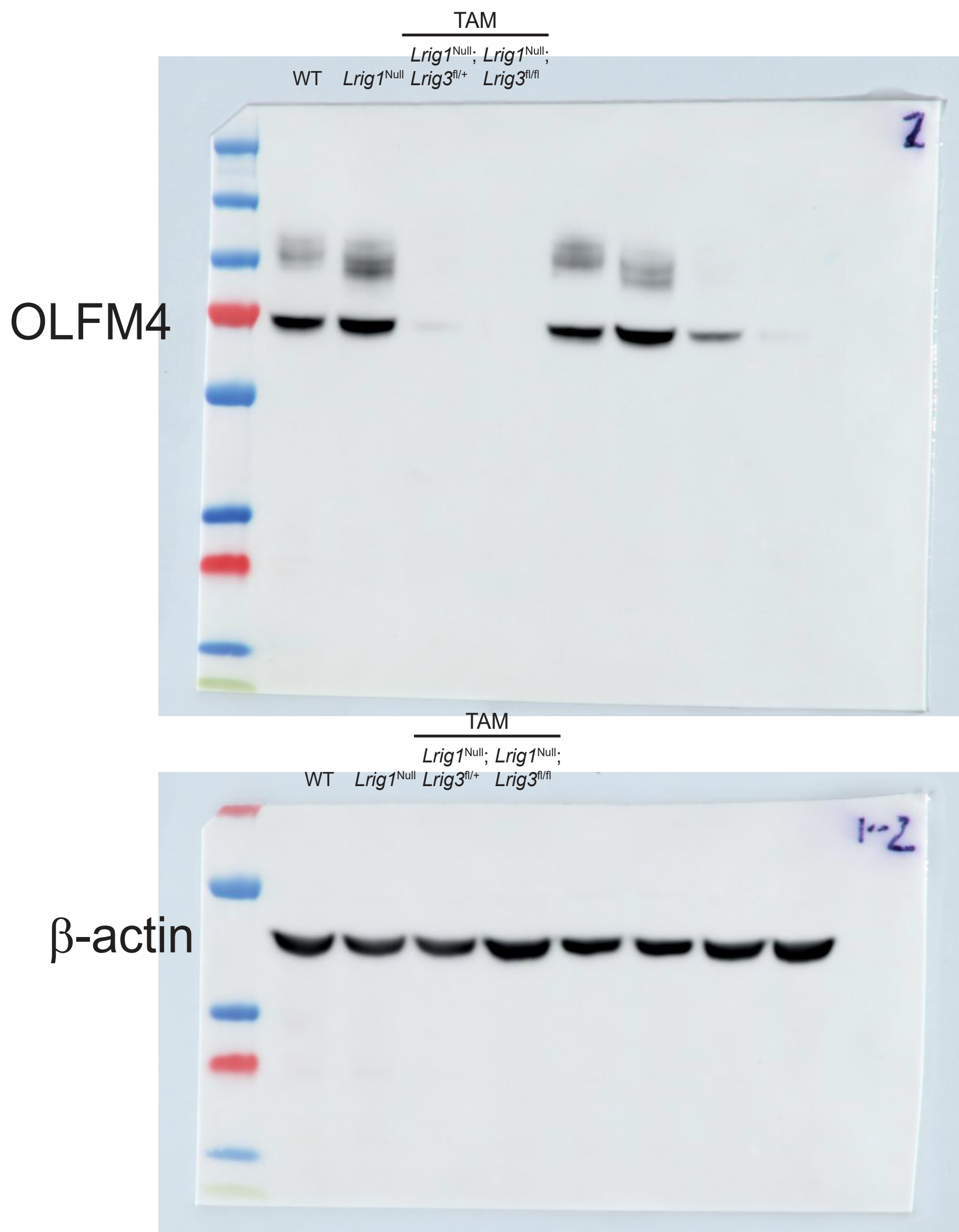

Figure 15B Westerns

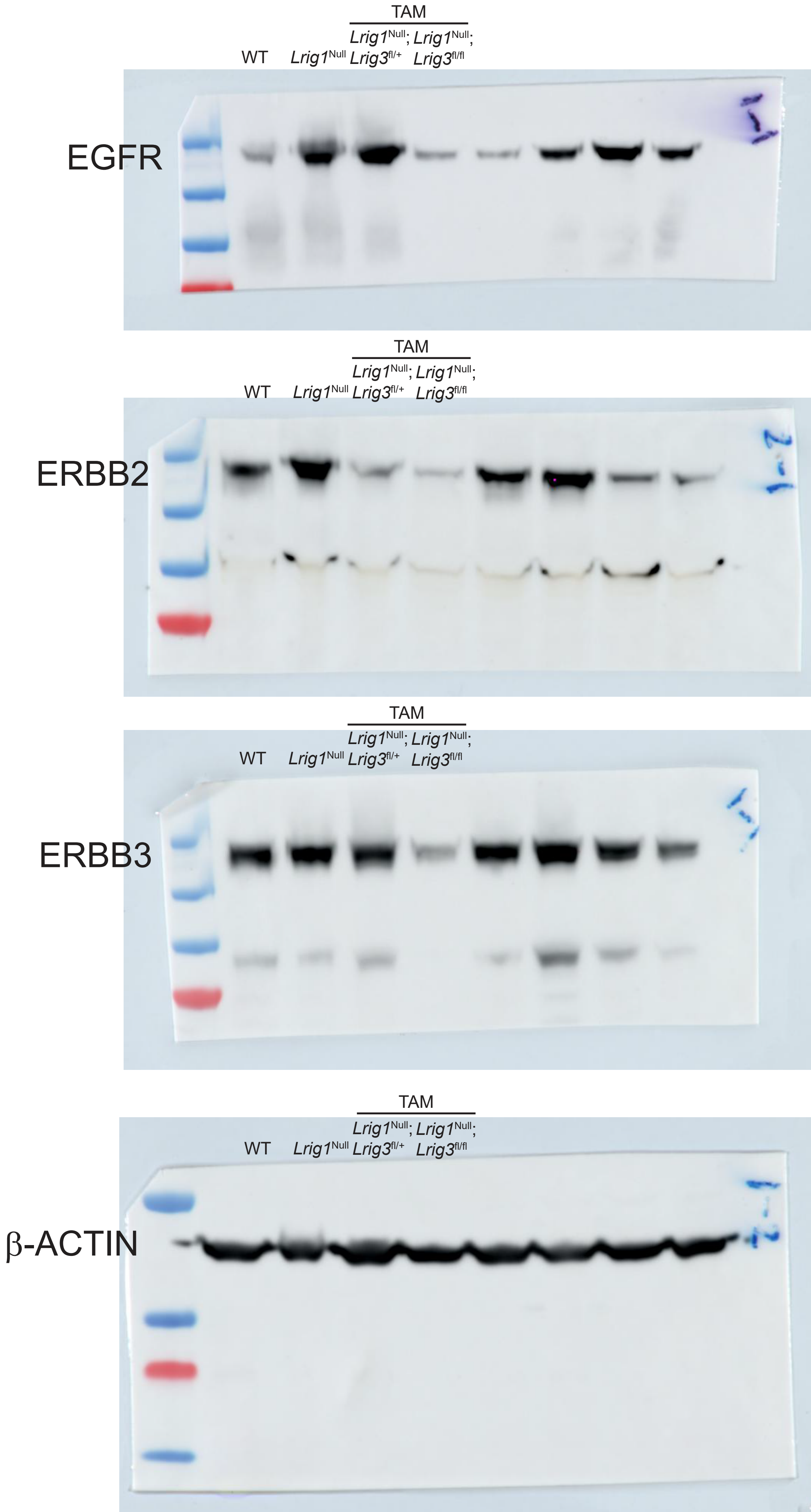

Supplement: Supplementary file 2 [file mmc2.pdf]
